# Prognostic and Therapeutic Implications of Cell Division Cycle 20 Homolog in Breast Cancer

**DOI:** 10.3390/cancers16142546

**Published:** 2024-07-15

**Authors:** Samia S. Messeha, Najla O. Zarmouh, Henrietta Maku, Sherif Gendy, Clement G. Yedjou, Rashid Elhag, Lekan Latinwo, Caroline Odewumi, Karam F. A. Soliman

**Affiliations:** 1 College of Science and Technology, Florida A&M University, Tallahassee, FL 32307, USA; samia.messeha@famu.edu (S.S.M.); clement.yedjou@famu.edu (C.G.Y.); rashid.elhag@famu.edu (R.E.); lekan.latinwo@famu.edu (L.L.); 2 College of Pharmacy & Pharmaceutical Sciences, Institute of Public Health, Florida A&M University, New Pharmacy Building, 1520 ML King Blvd, Tallahassee, FL 32307, USA; 3 Faculty of Medical Technology-Misrata, Libyan Ministry of Technical & Vocational Education, Misrata LY72, Libya; najlazar@yahoo.com; 4 Department of Pathology and Genomic Medicine, Houston Methodist Hospital, Houston, TX 77030, USA; ude.umaf@namilos.marak; 5 School of Allied Health Sciences, Florida A&M University, Tallahassee, FL 32307, USA; sherif.gendy@famu.edu

**Keywords:** CDC 20, breast cancer, TNBC, metastasis, immunomodulator

## Abstract

**Simple Summary:**

In breast cancer (BC), the triple-negative breast cancer subtype, the upregulation of CDC20 is positively associated with cancer initiation, progression, metastasis, and chemotherapy resistance. The current functional enrichment analysis study demonstrated the significant association of CDC20 co-expressed genes with biological regulation and cellular processes. This study reveals a significant positive correlation between overexpressed CDC20 and tumor purity and many immune cells; the finding suggests that CDC20 plays a fundamental role in controlling tumor immunity and consequently influences BC prognosis. CDC20 deficiency led to decreased cell growth and metastasis, G2/M cell cycle arrest, and boosted the cytotoxic effects of paclitaxel treatment, which supports the current analysis. Developing natural and synthetic inhibitors of this oncogene is a promising approach to the therapeutic management of BC.

**Abstract:**

Cell division cycle 20 homolog (CDC20) is a well-known regulator of cell cycle progression. Abnormal expression of CDC20 leads to mitotic defects, which play a significant role in cancer development. In breast cancer (BC), CDC20 has been identified as a biomarker that has been linked to poor patient outcomes. In this study, we investigated the association of CDC20 with BC prognosis and immune cell infiltration by using multiple online databases, including UALCAN, KM plotter, TIMER2.0, HPA, TNM-plot, bc-GenExMiner, LinkedOmics, STRING, and GEPIA. The results demonstrate that BC patients have an elevated CDC20 expression in tumor tissues compared with the adjacent normal tissue. In addition, BC patients with overexpressed CDC20 had a median survival of 63.6 months compared to 169.2 months in patients with low CDC20 expression. Prognostic analysis of the examined data indicated that elevated expression of CDC20 was associated with poor prognosis and a reduction of overall survival in BC patients. These findings were even more prevalent in chemoresistance triple-negative breast cancer (TNBC) patients. Furthermore, the Gene Set Enrichment Analysis tool indicated that CDC20 regulates BC cells’ cell cycle and apoptosis. CDC20 also significantly correlates with increased infiltrating B cells, CD4+ T cells, neutrophils, and dendritic cells in BC. In conclusion, the findings of this study suggest that CDC20 may be involved in immunomodulating the tumor microenvironment and provide evidence that CDC20 inhibition may serve as a potential therapeutic approach for the treatment of BC patients. In addition, the data indicates that CDC20 can be a reliable prognostic biomarker for BC.

## 1. Introduction

Cancer is a prominent cause of death, and continuous efforts are required to raise the life expectancy of cancer patients [1]. By the end of 2024, an estimated 2,001,140 new cancer cases and 611,720 cancer deaths are expected to occur in the United States [2].

In women, breast cancer (BC) is the second most prevalent cancer worldwide, after lung cancer, and still the leading cause of cancer-related death [3,4]. According to the most recent cancer statistics, approximately 313,510 new cases and 42,780 death cases from BC are projected [2]. BC is a complicated disease with several histological and biological characteristics, clinical symptoms and behaviors, and therapeutic outcomes [5].

According to the most common biomarkers and clinicopathologic characteristics [5], BC has been classified into five subtypes, including luminal A and B, HER2-enriched, basal-like, and claudin-low [6,7,8]. Triple-negative breast cancer (TNBC) is a molecular subtype of BC where the three receptors estrogen (ER), progesterone (PR), and human epidermal growth factor receptor 2 (HER-2) are unexpressed [4]. The status of these receptors changes throughout tumor progression, which has profound implications for BC patients [9]. The receptor can be changed to both directions, negative or both. However, negative conversion is higher, leading to the increased malignant phenotype, progression, and metastasis [10,11].

In BC therapy, ER, PR, and HER-2 receptors are the primary targets for efficient treatments [3,12]. Therefore, TNBC is categorized as the most aggressive subtype with the worst prognosis [12]. Recurrence and extensive metastasis are hallmarks of TNBC [13,14]. The current approach for enhancing the survival of TNBC patients includes developing more efficient immunotherapies [15], exploring targeted therapeutic drugs, and optimizing the existing chemotherapy regimens [16,17]. Additional studies are needed to expand treatment options for patients with TNBC. In parallel, the field of cancer genomics is continuously growing to interrogate the cellular and molecular characteristics of TNBC tumors to facilitate tailoring treatment strategies such as immunotherapies or targeted therapies [18,19,20]. Poly (ADP-ribose) polymerases (PARP), receptor tyrosine kinase targets, programmed cell death protein 1 (PD-1) and its ligand (PD-L1), as well as the mitogen-activated extracellular signal-regulated kinase (MEK) and protein kinase B (AKT) pathways, are considered the most common current targets for TNBC therapy [21,22,23]. Hence, identifying protein biomarkers and cell signaling pathways associated with TNBC is a promising approach for diagnosis, prognosis, and boosting chemotherapy and patients’ survival.

The significance of biomarkers in diagnosing and treating patients with various cancers, particularly BC, is becoming increasingly significant [24]. Many biomarkers, such as IL21R [25], BIRC5 [26], SLC7A11 [27], PKM2 [28], PPP1R14A [29], and ZBTBA7 [30], as well as others, have been previously cited.

In cancer research, biomarkers regulating cell cycle pathways are a critical area of study. While the cell cycle could be halted at different phases, attention was mainly directed to proteins controlling the S-phase and mitosis phases, where DNA is synthesized and segregated [12].

As a marker for protein destruction, cell division cycle 20 (CDC20) plays a crucial role in regulating the cell cycle transition from metaphase to anaphase and then exits to interphase during mitosis. At the end of M-phase, when the spindle assembly checkpoint component is established, CDC20 activates anaphase-promoting complex/cytoplasm (APC/C), a ubiquitin ligase necessary for inducing chromatid separation and proceeding cell cycle into the anaphase [31,32]. APC/C, in turn, aids in the degradation of cyclins by tagging them with ubiquitin for proteasomal degradation. In eukaryotic organisms, CDC20 expression fluctuates over different cell cycle phases [33]. CDC20 mRNA levels are elevated in cells arrested at the G1 phase, decline during the S phase, and rise again during G2 and mitosis [33]. Accordingly, the level of CDC20 protein increases during the S-phase, peaks throughout mitosis, and lowers once mitosis terminates [32,33,34]. Understanding the regulation of CDC20 levels is the key to precisely interpreting cell cycle events [34]. Therefore, impaired CDC20 function may terminate mitotic arrest and lead to the initiation and progression of cancer [35,36,37].

Many studies have shown that the oncogene CDC20 is significantly overexpressed in most tumor types, including BC [37,38,39,40,41], and its overexpression has been suggested as a biomarker of poor outcomes [37,38,42,43]. Another cohort study emphasized the correlation between elevated levels of CDC20, the aggressive development of BC, and poor outcomes [44]. Thus, it was suggested that targeting CDC20 could be a potential therapeutic strategy for tumors with high CDC20 expression levels [43]. The development of natural and synthetic CDC20 inhibitors is a novel avenue that should proceed to clinical studies to treat tumors [43].

In the current study, we established a thorough investigation of CDC20 in BC, focusing additional attention on TNBC. We determined the significance of CDC20 by evaluating differential expressions, clinical survival prognosis, genetic alteration, immune infiltration landscape, and putative signaling pathway. We also explored the underlying tumorigenesis and tumor suppression mechanism across different cancer types. The expression of CDC20 in the other cancer types was also addressed using the information available on various databases. Altogether, this inclusive analysis will shed light on the behavior of CDC20 in BC and evaluate the rationale of CDC20 inhibition as a potential therapeutic approach for treating BC patients with CDC20 overexpression.

## 2. Materials and Methods

**Figure 1:**
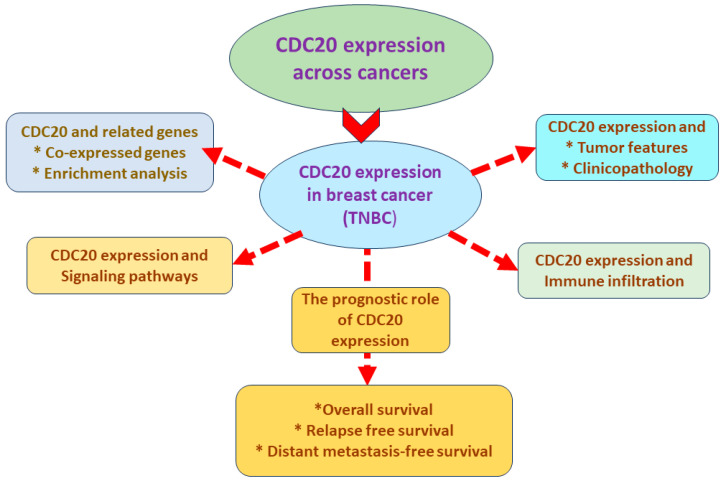
Summary of the established analyses.

A comprehensive analysis was conducted, using different web tools as summarized in Figure 1.

### 2.1. UALCAN; CDC20 Transcript and Protein Expressions in Tumors, BC Subtypes, and Normal Cells

For our investigation, UALCAN (http://ualcan.path.uab.edu, accessed on 4 March 2023), is a common web source for broad analysis of the large cancer genomics dataset available. The Cancer Genome Atlas (TCGA) data of UALCAN were also used. In this study, we employed UALCAN to validate the relative gene and protein levels of CDC20 across tumor and normal samples and in different BC subtypes at various stages [45].

### 2.2. KM Plotter; CDC20 Prognostic Value in BC and TNBC Patients

The Kaplan–Meier (KM) Plotter (http://kmplot.com/analysis/, accessed on 4 March 2023), is a web tool that checks the effect of 54,675 genes on survival using 5143 clinical breast cancer samples. The association between a clinical biomarker (our parameter, CDC20) and survival can be visualized by the generated KM plot, in which patients are assigned into groups according to the parameters. We applied this tool to show the prognostic value of CDC20 in relapse-free survival (RFS), overall survival, and distant metastasis-free survival [39]. Values were calculated simultaneously [46,47,48]. Receiver operating characteristic (ROC) analyses were used to evaluate the performance (https://rocplot.org/site/treatment, accessed on 4 March 2023) [49].

### 2.3. TIMER2.0; CDC20 Transcript Expression in Tumor and Normal Tissue

Tumor Immune Estimation Resource TIMER2.0 (cistrome.org, accessed on 4 March 2023), database analysis was used for this investigation. For gene expression analysis, we entered CDC20 into the “GENE_DE” module, exploring associations between gene expression and tumor features in TCGA [50,51,52]. This database allowed us to generate more of the significant difference between CDC20-overexpressed tumors and normal tissues.

### 2.4. HPA Database; CDC20 Protein Expression and Associated Gene Clusters in BC and Normal Tissues

We accessed the immunohistochemistry (IHC) of the Human Protein Atlas (HPA) Database (https://www.proteinatlas.org, accessed on 4 March 2023) [46] to ascertain if the CDC20 protein was differentially expressed in normal and BC tissues. This database provided qualitative analysis of CDC20 protein expression of tumor pathology relative to normal tissues. (https://www.proteinatlas.org/ENSG00000117399-CDC20/pathology/breast+cancer, accessed on 4 March 2023). In addition, HPA RNA-seq data were used to identify gene clusters associated with the expression of CDC20 in BC (accessed on 20 October 2023).

### 2.5. TNM-Plot; CDC20 Transcript Expression in BC and Normal Tissues

To compare the expression of CDC20 in tumors to normal tissues, we accessed the Tumor Normal-Metastatic (TNM)-plot (https://tnmplot.com/analysis/, accessed on 4 March 2023). This tool provided boxplot analysis for the CDC20 gene using RNA-Seq-based data. The normal samples presented non-cancerous patients and further pediatric tissues [53].

### 2.6. Bc-GenExMiner Database; CDC20 Transcript Expression among BC Subtypes

The most updated Breast Cancer Gene-Expression Miner v5.0 (bc-GenExMiner v5.0) database provided several types of analyses: correlation, expression, and prognosis (http://bcgenex.ico.unicancer.fr/BC accessed on 4 March 2023). The DNA microarrays allowed us to assess the association of CDC20 gene expression with overall survival (OS), disease-free survival (DFS), and distant metastasis-free survival (DMFS) in all TNBC patients, in addition to four more subtypes: basal-like immune-activated (BLIA), basal-like immune-suppressed (BLIS), luminal androgen receptor (LAR), mesenchymal-like immune-activated (MLIA) [54,55,56]. Also, the muTarget analysis tool was used to link CDC20 gene expression changes with mutation status in BC tumors to examine the potential of CDC20 as a biomarker in different BC subtypes.

### 2.7. TIMER2.0; BC CDC20 Expression and Immune Infiltration Analysis

The the online resource TIMER2.0 allowed us to establish a systematic analysis of immune infiltrates from TCGA (https://cistrome.shinyapps.io/timer/, accessed on 4 March 2023) [37]. TIMER2.0 speculates the abundance of tumor-infiltrating immune cells from the gene expression profile [38]. The gene module allows us to select CDC20 and visualize the correlation of its expression with immune infiltration levels in BC. We investigated the correlation of CDC20 expression with the abundance of immune infiltrates, including B cells, CD4+ T cells, CD8+T cells, neutrophils, macrophages, and dendritic cells in BC. The somatic copy number alterations (SCNAs) module was used to compare tumor infiltration levels among tumors with different SCNAs for a given gene. Genomic Identification of Significant Targets in Cancer (GISTIC 2.0) was used to identify genes targeted by SCNAs in cancer. The P-values and partial correlation values were obtained using a purity-adjusted Spearman’s rank correlation test. Heatmaps and scatter diagrams were also obtained. Box plots were presented to show the distributions of each immune subset at each copy number status in BC. The infiltration level for each SCNA category is compared with the normal using a two-sided Wilcoxon rank sum test.

### 2.8. LinkedOmics, GO, KEGG, and PANTHER; CDC20 Attributes and Related Genes

The LinkedOmics database (http://www.linkedomics.org, accessed on 4 March 2023), an extensive online platform, was used to access, analyze, and compare 32 cancer-related data and ten Clinical Proteomics Tumor Analysis Consortium (CPTAC) cancer cohorts. To identify associated genes, we employed two analytical modules: the LinkFinder module was used to search for gene attributes and functional contexts that are associated with CDC20, and the Link Interpreter module was used to derive biological insights from the association results, followed by biological pathways analysis using Gene Ontology (GO) annotations on the specific genes co-expressed with CDC20 [57]. Under the Link Interpreter module, Gene Set Enrichment Analysis (GSEA) was selected to generate the GO and Kyoto Encyclopedia of Genes and Genomes (KEGG) biological pathways data to understand CDC20-related biological pathways in the cell biological system. Spearman’s correlation analysis was established to examine significance using the false discovery rate (FDR) and the *p*-value [57]. Further, the PANTHER classification system (http://www.pantherdb.org, accessed on 4 March 2023), a comprehensive platform for analyzing gene function on a genome-wide scale, was used for related genes and proteins functional classification in CDC20 expressed BC.

### 2.9. STRING; CDC20 Related Gene Enrichment Analysis

We used the Search Tool for the Retrieval of Interacting Genes/Proteins (STRING): functional protein association networks (string-db.org) [58]. Under the protein name (CDC20), we set the following parameters as previously mentioned [59]: the organism, Homo sapiens, meaning of network edges, confidence; active interaction sources, text mining; experiments, databases; co-expression, gene fusion, and co-occurrence. We also set the minimum required interaction score at low confidence of 0.150 and no more than 50 interactors as the maximum number of interactors. We obtained the top 50 CDC20-related evidence-based proteins and corresponding network diagrams. To detect genes with an expression pattern analogous to CDC20 in different cancers, we entered the gene (CDC20) and top 100 in the Expression Analysis Similar Gene Detection (EASGD) module of the GEPIA2 website. We selected all TCGA tumor data sets for this analysis. We selected the top five genes from the list and entered the gene CDC20 and the top five genes in the expression analysis correlation analysis module of GEPIA2. Further, we used the Exploration Gene Corr module of TIMER2.0 to acquire the heatmap data for the top five genes and CDC20 in various cancer types. Pearson correlation coefficients (R values) and *p*-values were obtained, and Spearman’s rho value was generated to indicate the degree of correlation.

## 3. Results

### 3.1. Analysis of CDC20 mRNA and Protein Expressions in BC Compared to Normal Tissues

**Figure 2:**
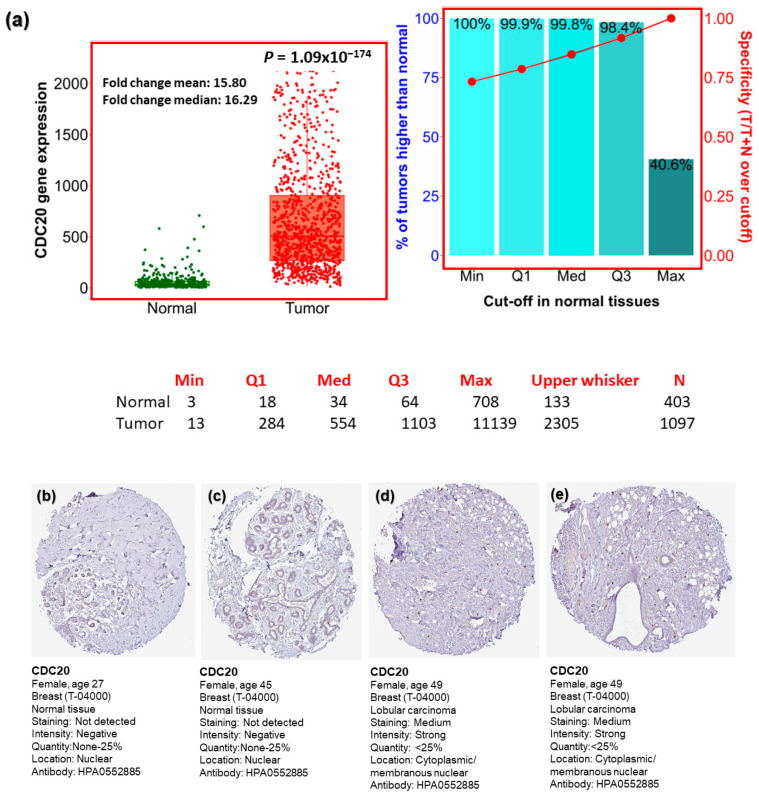

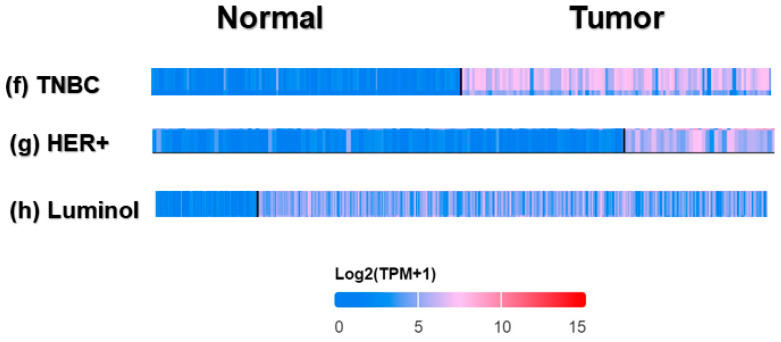
CDC20 transcript and protein expression in breast cancer (BC). (**a**) Plots present the expression of CDC20 in normal and BC tissues. (**b**–**e**) CDC20 protein IHC images in normal tissue and lobular carcinoma. (**f**–**h**) The heat map profile for CDC20 expression in TNBC compared to other BC subtypes (epidermal growth factor receptor 2 positive (HER2+) and luminol. UALCAN analysis on TCGA and HPA was used. IHC; immunohistochemistry.

Our comprehensive BC investigation of the TCGA dataset showed a substantial upregulation (*p* = 1.09 × 10^−174^) in CDC20 mRNA expression in tumors compared to normal tissues (Figure 2a). Compared to normal breast tissues, a mean fold change of 15.80 and a median fold change of 16.29 were found in BC cells. Furthermore, the Human Protein Atlas was used to validate the upregulated expression of the CDC20 gene using IHC images for CDC20 protein in tumor and normal breast tissue. The normal tissues showed “Not detected” staining with “negative” intensities (Figure 2b,c), while BC tissues demonstrated “medium staining” with “strong” intensities in the cytoplasm and around the nuclear membrane (Figure 2d,e). These qualitative IHC images are consistent with the upregulation of CDC20 protein in BC patients obtained from TCGA. Furthermore, the heat map profile from ULCAN highlighted the highest expression of CDC20 in TNBC, followed by HER2+ and luminol (Figure 2f–h).

### 3.2. CDC20 Gene Expression Based on Hormone and BC1/2 Status

**Figure 3:**
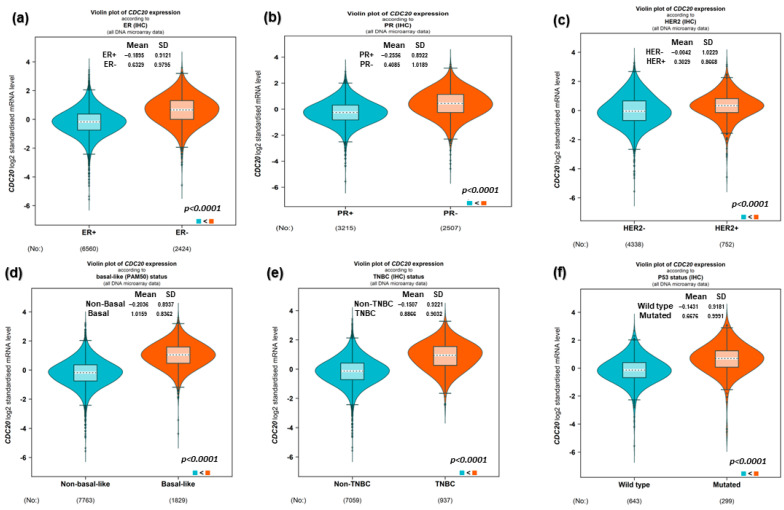
mRNA expression of CDC20 in BC based on hormone classification and BC1/2 mutation. (**a**–**c**) The CDC20 expression was upregulated in patients diagnosed with ER−, PR−, and HER- compared to patients with ER+, PR+, and HER2+. (**d**,**e**) A high expression of CDC20 was also found in basal-like and TNBC patients, compared to non-basal and non-TNBC patients. (**f**) Upregulated CDC20 mRNA was found in BC1/2 mutated patients compared to wild-type patients. UALCAN analysis of the TCGA database was used. ER; estrogen receptor, PR; progesterone receptor.

The log2 standardized mRNA expression of CDC20 was investigated to evaluate factors such as the expression of the hormone receptors and BC1/2 mutation (Figure 3). As shown in Figure 3a–c, the expression of CDC20 was significantly upregulated (*p* < 0.0001) in patients diagnosed with ER−, PR−, and HER− compared to ER+, PR+, and HER2+ patients, respectively. These findings were consistent with the highly upregulated expression of CDC20 in patients with both basal and TNBC subtypes compared to non-basal and non-TNBC (*p* < 0.0001, Figure 3d and e, respectively). Furthermore, BC patients with mutated BC1/2 have shown high expression of the CDC20 mRNA compared with the wild-type diagnosed patients (*p* < 0.0001, Figure 3f).

### 3.3. CDC20 mRNA Expression in Various Clinicopathological Features in BC

**Figure 4:**
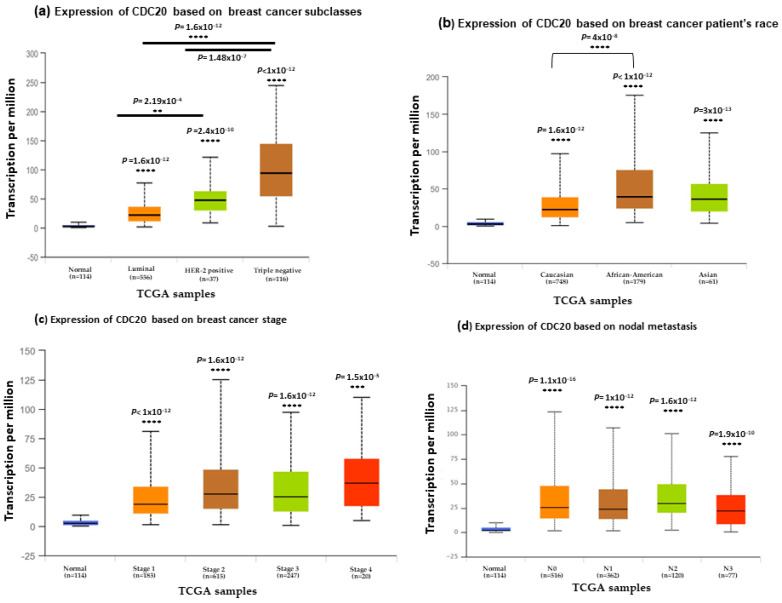
CDC20 transcript expression in four common clinicopathological features of BC cell samples (**a**) expression of CDC20 in different BC subtypes. (**b**) CDC20 expression in different racial groups of BC. (**c**) CDC20 expression in various stages of breast cancer compared to the normal breast tissue. (**d**) Upregulated level of CDC20 across various stages of metastatic nodal. All graphs are generated through UALCAN analysis of the TCGA database. *p*-values; ** *p* < 0.01, *** *p* < 0.001, and **** *p* < 0.0001.

We investigated the expression of CDC20 in several clinicopathological features in BC, including subtype, race, stage, and nodal metastasis (Figure 4). Data from TCGA and UALCAN showed a significantly elevated level of CDC20 (*p* < 1 × 10^−12^) in the most aggressive TNBC subtype patients (Figure 4a) patients with African American origin compared to the Caucasian and Asian racial groups (*p* < 1 × 10^−12^, Figure 4b). The four stages of BC progression showed significant CDC20 overexpression. Meanwhile, stage 1 revealed the most significant upregulated level of CDC20 (*p* < 1 × 10^−12^, Figure 4c) compared to the normal breast tissue. Consistently, this upregulation in CDC20 expression was also in parallel to its upregulation in the four metastatic nodals (N0, N1, N2, and N3), giving the highest level at the first three nodal stages and an outstanding significance in N1 (*p* < 0.0001, Figure 4d). In brief, these results confirmed high expression in CDC20, particularly in TNBC, the African American race, at all stages of grades and nodal metastasis.

### 3.4. The Impact of CDC20 Expression on BC Patients’ RFS and Complete Pathological Response (CPR)

**Figure 5:**
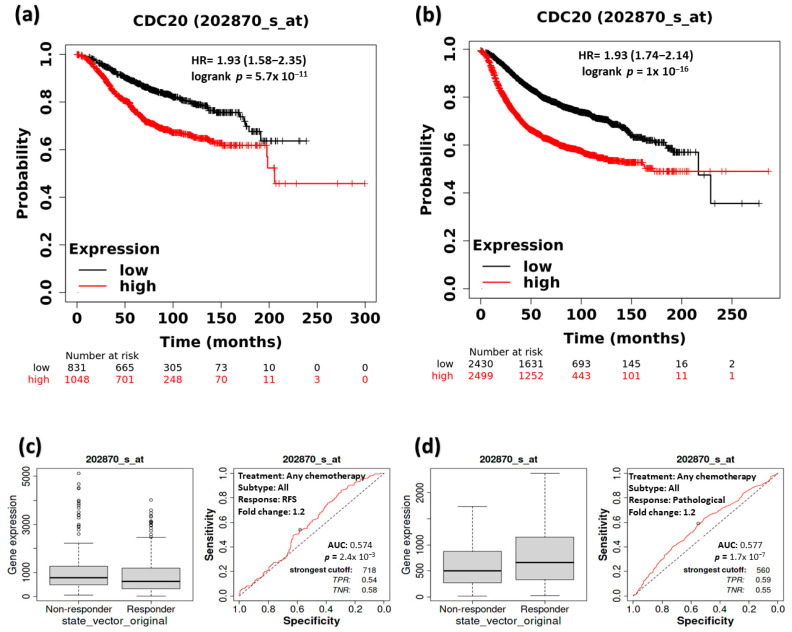

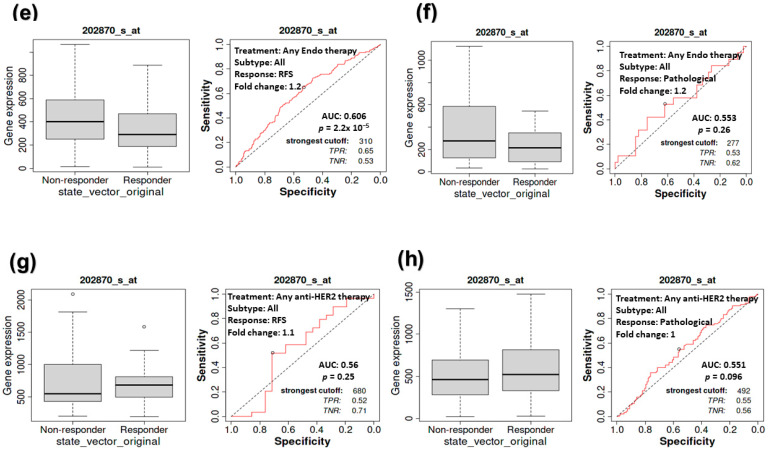
The relationship between CDC20 expression, RFS, and CPR in BC patients. At low and high expression of CDC20, the median RFS rate of systemically (**a**) untreated patients and (**b**) treated patients. RFS and CPR in patients receiving any form of chemotherapy (**c**,**d**), any endo therapy (**e**,**f**), or any anti-HRE2 therapy (**g**,**h**). Data were presented as a Kaplan–Meier (KM) plot HR, hazard ratio, RFS, relapse-free survival, CPR, and complete pathological response.

KM survival plot and ROC analyses were employed to ascertain whether CDC20 overexpression interferes with overall survival and CPR) in BC patients (Figure 5). The RFS median rate in both systematically untreated vs. systematically treated patients was measured at low and high expression of CDC20 (Figure 5a,b). In systematically untreated patients (Figure 5a), the upper quartile RFS rate of the high expression group was 63.6 months compared to 169.2 in its counterpart with low CDC20 expression (HR, 1.93; CI, 1.58–2.35; *p* = 5.7 × 10^−11^). The systematically treated BC patients (Figure 5b) showed a median RFS of 171.43 months in the highly expressed CDC20 group. Meanwhile, the low expression group had a higher median RFS of 216.66 months (HR, 1.93; CI of 1.74–2.14; *p* ˂ 1 × 10^−16^). That indicates that patients with higher CDC20 expression have poorer prognosis, regardless of their systematic treatment.

Furthermore, we explored the RFS in comparison with the complete pathological response (CPR for all BC subtypes that received any chemotherapy (Taxane, FAC, Ixabepilone, Anthracycline, FEC, or CMF), any endocrine therapy, or any anti-HER2 therapy (Figure 5c–h). Notably, a significant association between the decrease in CDC20 expression and the increase in RFS was found in patients receiving any form of chemotherapy (*p* = 2.4 × 10^−3^) or any endocrine therapy (*p* = 2.2 × 10^−5^) (Figure 5c,e). However, significant CPR (*p* = 1.7 × 10^−7^) was only exhibited in the chemotherapy-treated group (Figure 5d). In contrast, RFS or CPR was not statistically significant (*p* = 0.26 and 0.096, respectively), for patients who received anti-HRE2 therapy (Figure 5g,h). BC patients with higher expression of CDC20 have shown a worse prognosis compared with their counterparts with low levels of CDC20.

### 3.5. The Impact of CDC20 Expression on CPR in TNBC Subtype Patients

**Figure 6:**
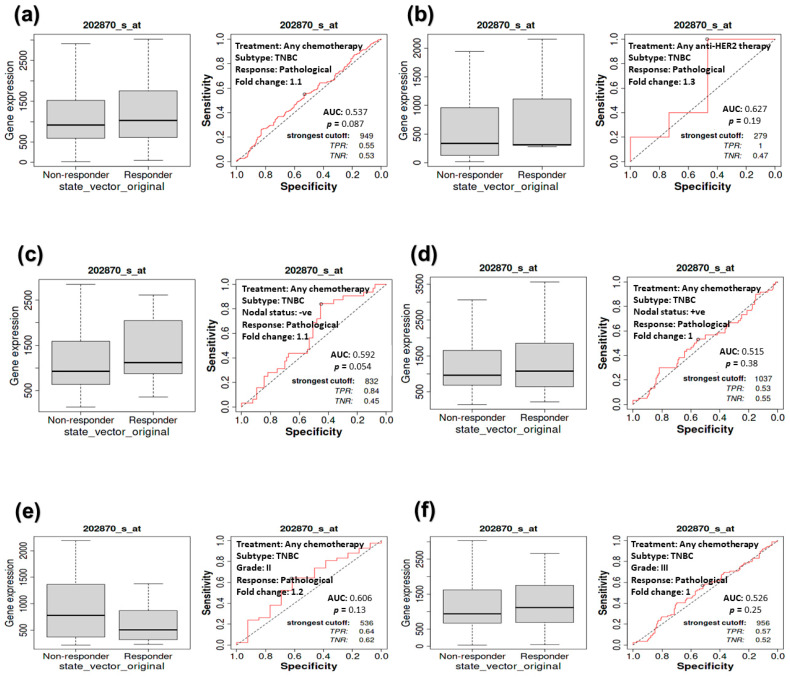
The relationship between CDC20 expression and CPR in TNBC patients. Compared with the non-receiving group, no significant relationship was found between CDC20 expression and pathological response in patients receiving (**a**) chemotherapy (*p* = 0.087) or (**b**) anti-HER2 therapy (*p* = 0.19). No significant difference was exhibited between responder vs. non-responder TNBC patients in the following categories: nodal status (**c**) negative (*p* = 0.054) or (**d**) positive (*p* = 0.38), (**e**) grade II (*p* = 0.13) or (**f**) grade III (*p* = 0.25).

The KM plot of survival was used to examine the relationship between CDC20 expression and CPR, specifically in TNBC. For further validation, we performed ROC analysis to determine the predictive biomarkers in BC. CDC20 mRNA overexpression in TNBC subtypes was linked to a worse prognosis. From the TNBC patients’ data analysis, no significant difference (*p* > 0.05) was found between the responder to therapy compared to the non-responder, respectively, in the following groups: TNBC patients who received any chemotherapy (Figure 6a, median of 1028, *n* = 5016 vs. 919, *n* = 4492), or anti-HER2 therapy (Figure 6b; median of 318, *n* = 2157 vs. 337, *n* = 1940), TNBC patients with negative nodal (Figure 6c, median of 1118, *n* = 3858 vs. 932, *n* = 3680) or positive nodal (Figure 6d; median of 1076, *n* = 5016 vs. 966, *n* = 4492), as well as grade II (Figure 6e; median of 507, *n* = 2560 vs. 776, *n* = 3680) and grade III (Figure 6f; median of 1113, *n* = 5016 vs. 934, *n* = 4492). In other TNBC patient groups who received any of the previously mentioned therapies or at the mentioned states of the disease or stage, no significant relationship was found between CDC20 expression and CPR (Figure 6a–f; *p* > 0.5). (https://rocplot.org/site/treatment) [28].

### 3.6. The Association between CDC20 Expression and RFS in TNBC Patients

**Figure 7:**
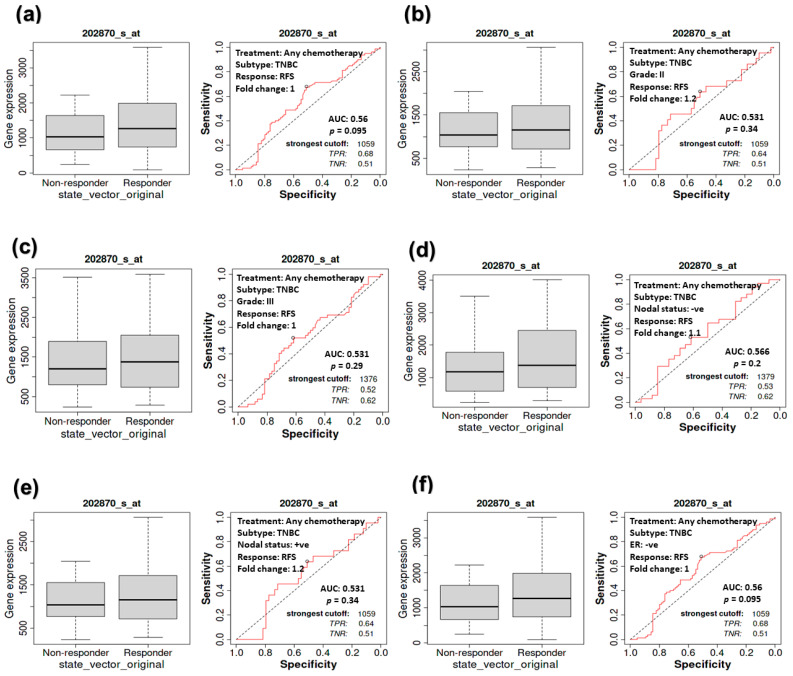
The relationship between CDC20 expression and RFS in TNBC patients. No significant correlation was detected between CDC20 expression and RFS in the tumors of (**a**) TNBC subtype, (**b**) grade II, (**c**) grade III, (**d**) nodal positive, (**e**) nodal negative, or (**f**) ER-subtype, regardless of chemotherapy status.

Investigating the RFS of TNBC patients receiving chemotherapy (Figure 7) showed similar behavior in the counterpart CPR study (Figure 6). A consistent, non-significant correlation (*p* = 0.095) between CDC20 expression and RFS was exhibited in TNBC patients, whether or not they received chemotherapy (Figure 7a). No significant correlation was found in the other groups of TNBC patients with grade II (*p* = 0.34, or III (*p* = 0.29) tumors (Figure 7b,c), with negative (*p* = 0.20) or positive (*p* = 0.34) nodal status (Figure 7d,e) or ER-tumors (*p* = 0.095, Figure 7f). These consistent findings demonstrate the role of CDC20 overexpression in TNBC aggressiveness.

### 3.7. Expression of CDC20 in Relation to DFS, OS, and DMFS in TNBC Patients

**Figure 8:**
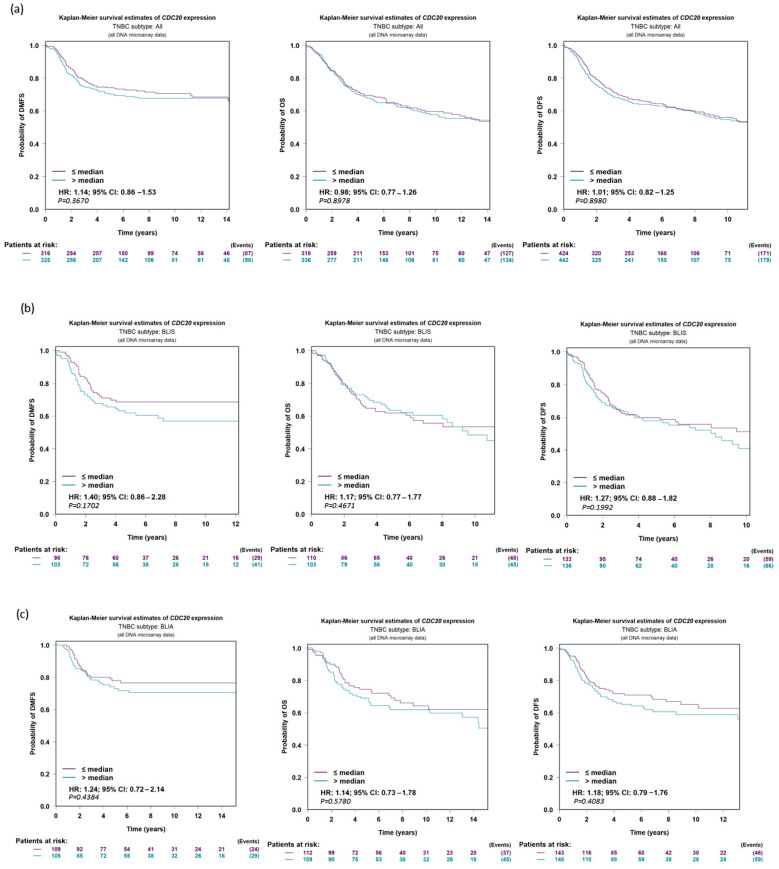

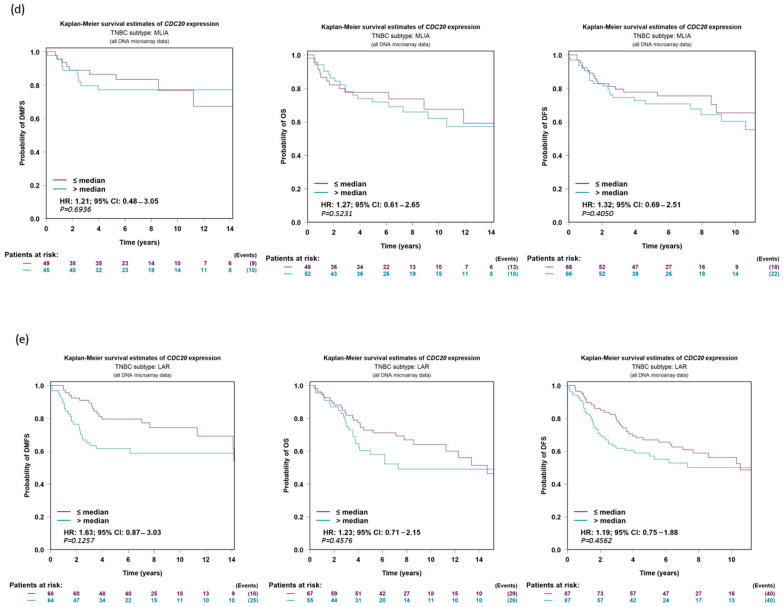
CDC20 expression in TNBC in relation to DFS, OS, and DMFS. There is no significant difference evident between low and high expressions of CDC20 in relation to survival in (**a**) all TNBC subtypes or individual subtypes, (**b**) BLIS, (**c**) BLIA, (**d**) MLIA, or (**e**) LAR. The UALCAN data were presented on a KM plot. A DNA microarray median probe univariate Cox analysis with a CI of 95% was used. DFS; disease-free survival, OS; overall survival, DMFS; distant metastasis-free survival, BLIS; basal-like immune-suppressed, BLIA; basal-like immune-activated, MLIA; mesenchymal-like immune-activated, LAR; luminal androgen receptor.

The data analysis program bc-GenExMiner v5.0 was used to establish a prognostic analysis of IHC subtypes of TNBC. The UALCAN data presented on KM plot did not show any significant difference (*p*-value > 0.10) between low and high CDC20 expression in relation to DMFS, OS, or DFS in the five following groups of TNBC: all TNBC subtypes (Figure 8a), basal-like immune-suppressed (BLIS, Figure 8b), basal-like immune-activated (BLIA, Figure 8c), mesenchymal-like immune-activated (MLIA, Figure 8d), and luminal androgen receptor (LAR, Figure 8e). Thus, the change in overexpression of CDC20 is not associated with the prognosis in TNBC histochemical subtypes.

### 3.8. Association of Ten Commonly Mutated Genes with CDC20 mRNA Expression in BC

**Figure 9:**
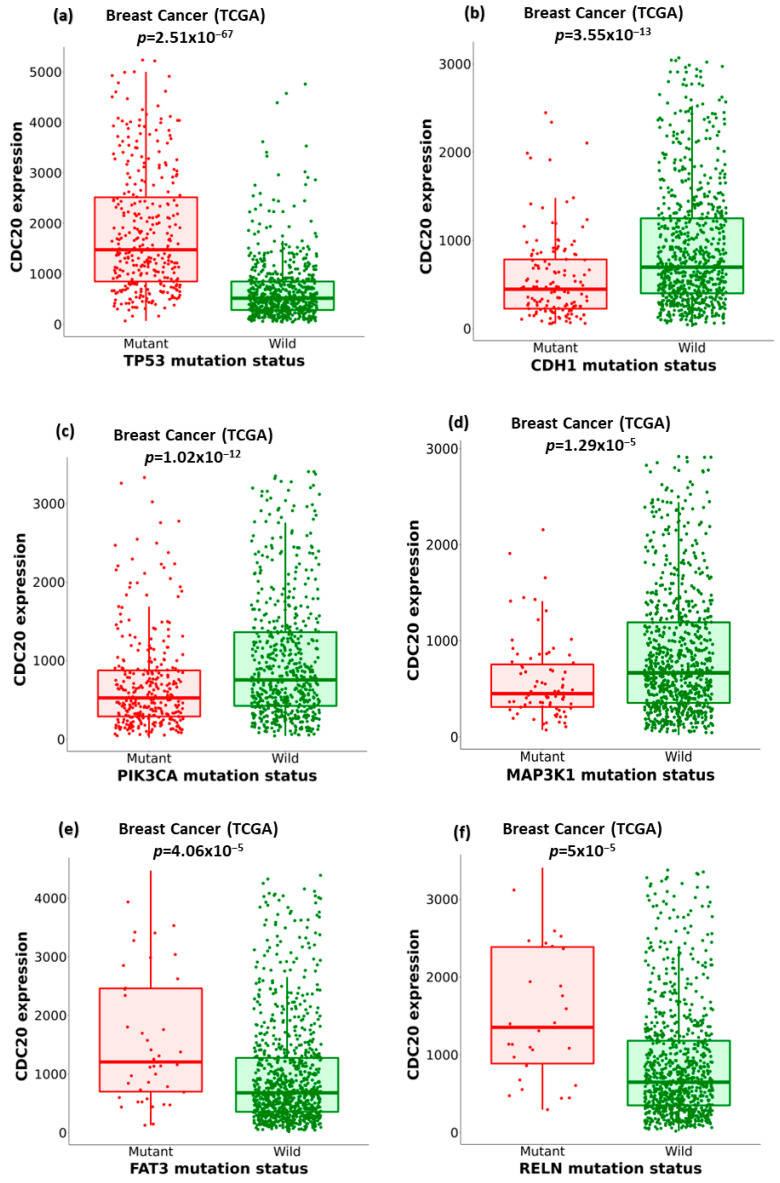

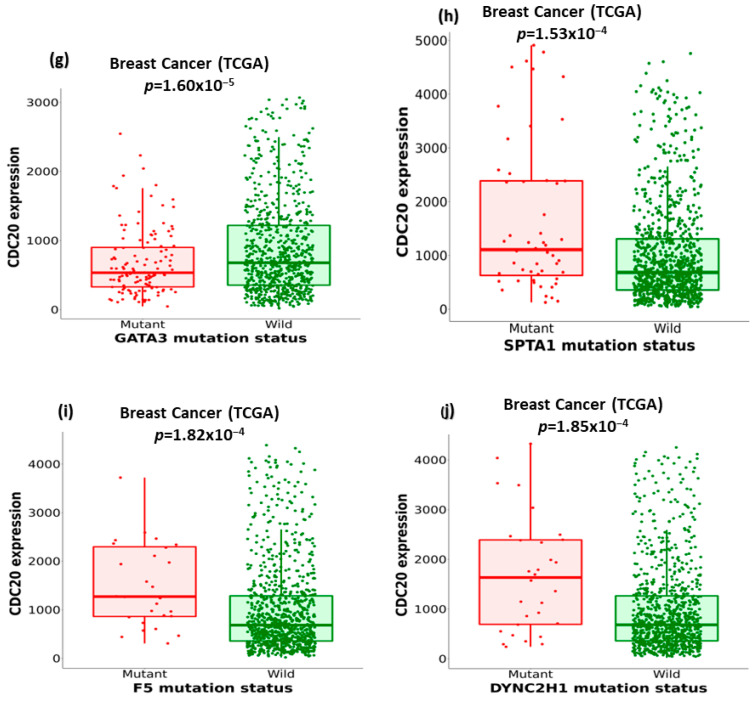
The relationship between CDC20 expression and critical gene mutations in BC, other than BC1/2. (**a**–**j**) Boxplots comparing the expression of CDC20 in the wild type BC to its expression in the mutation status of the most commonly changed genes: TP53, tumor protein P53; CDH1, cadherin-1; PIK3CA, phosphoinositide-3-kinase, catalytic, alpha polypeptide; MAP3K1, mitogen-activated protein kinase 1; FAT3, FAT atypical cadherin 3, RELN, reelin; GATA3, GATA binding protein 3; SPTA1, spectrin alpha, erythrocytic 1; F5, coagulation factor V; DYNC2H1, cytoplasmic dynein 2 heavy chain 1.

We compared the expression of CDC20 in wild-type BC to its expression in ten of the commonly mutated forms of proteins and genes other than BC1/2 (Figure 9). The investigation included tumor protein P53 (TP53), cadherin-1 (CDH1), phosphoinositide-3-kinase, catalytic, alpha polypeptide (PIK3CA), mitogen-activated protein kinase-1 (MAP3K1), FAT atypical cadherin 3 (FAT3), reelin (RELN), GATA binding protein 3 (GATA3), spectrin alpha, erythrocytic 1 (SPTA1), coagulation factor V (F5), and cytoplasmic dynein 2 heavy chain 1 (DYNC2H1) (Figure 9a–j). In BC, the mutated TP53, FAT3, RELN, SPTA1, F5, and DYNC2H1 genes showed overexpression of CDC20. Meanwhile, tumors with CDH1, PIK3CA, MAP3K1, and GATA3 genes were accompanied by downregulation of CDC20.

**Figure 10:**
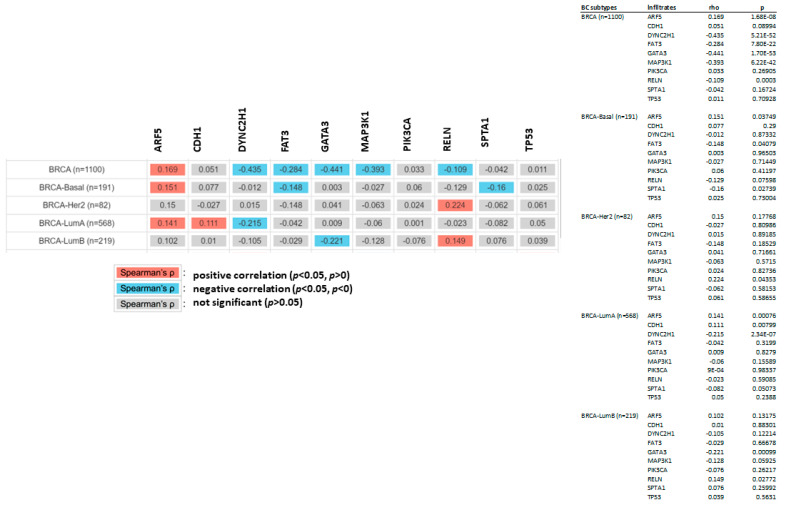
The relationship between mutated genes and CDC20 expression in different subtypes of BC. The positive (red) and negative (blue) correlation between the commonly mutated genes and CDC20 in all BC subtypes: BRICA-BASAL, BRICA-HER2, BRICA-LUMA, and BRICA-BUMB using the non-parametric test Spearman’s rho. *p* > 0.05 is a non-significant difference.

The relationship between the previously mentioned genes and CDC20 expression was further investigated in different subtypes of BC using the non-parametric test, Spearman’s rho. According to the rho value, the association level was categorized into positive (rho = +ve) or negative (rho = −ve), as highlighted in Figure 10. The analysis indicated different correlation patterns between the mutated genes and CDC20 expression. In all BC subtypes, the expression of five genes, DYNC2H1, FAT3, GATA3, MAP3K1, and RELN mRNA, were negatively correlated with CDC20 overexpression in BC. Furthermore, this negative correlation was also found between CDC20 overexpression and specific mutated genes, such as FAT3 and SPTA1 in the BC basal subtype, DYNC2H1 in the BC LumA, and GATA3 in BC LumB. CDC20 expression was positively correlated with mutated ARF5 in all BC subtypes, CDH1 in BC LumA, RELN in BC Her2, and BC LumB. The impact of these mutated genes on CDC20 expression is summarized in Table 1.

### 3.9. Analysis of GO and KEGG Biological Pathways of the Genes co-Expressed with CDC20 in BC

**Figure 11:**
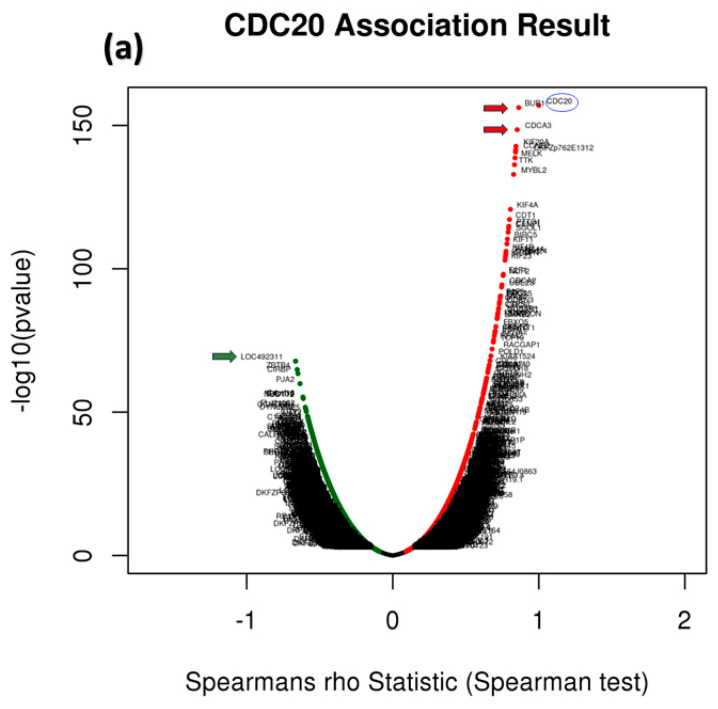

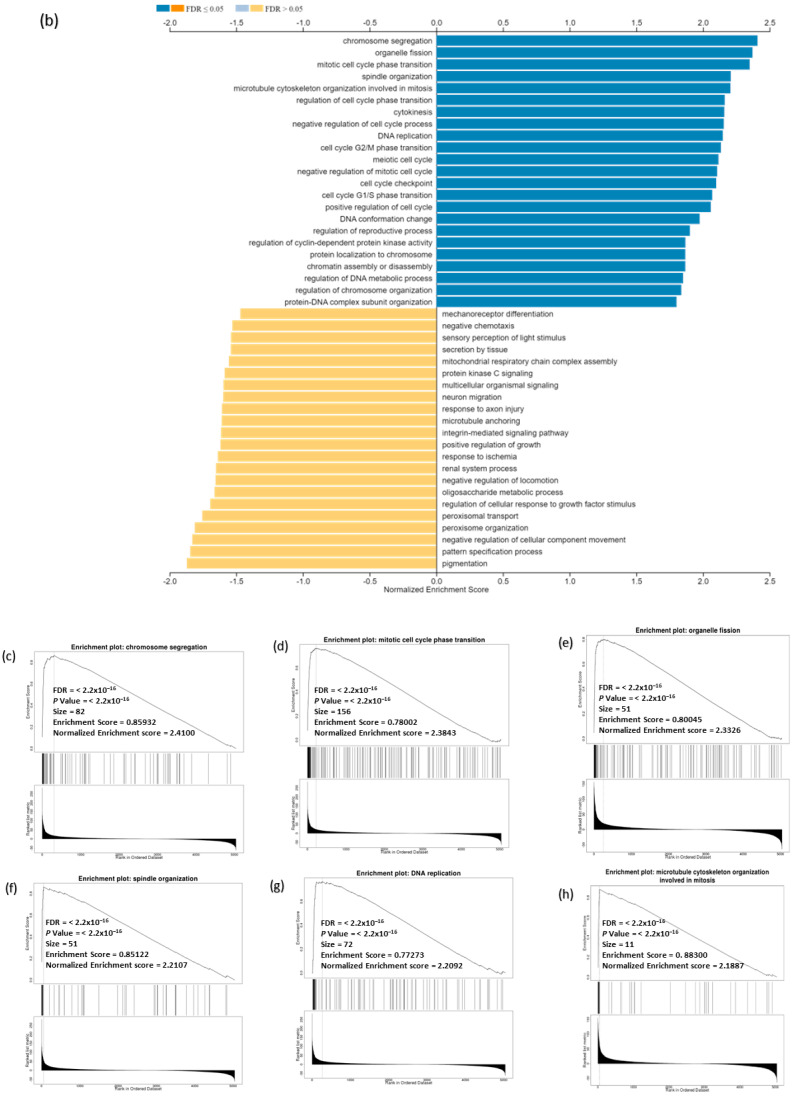

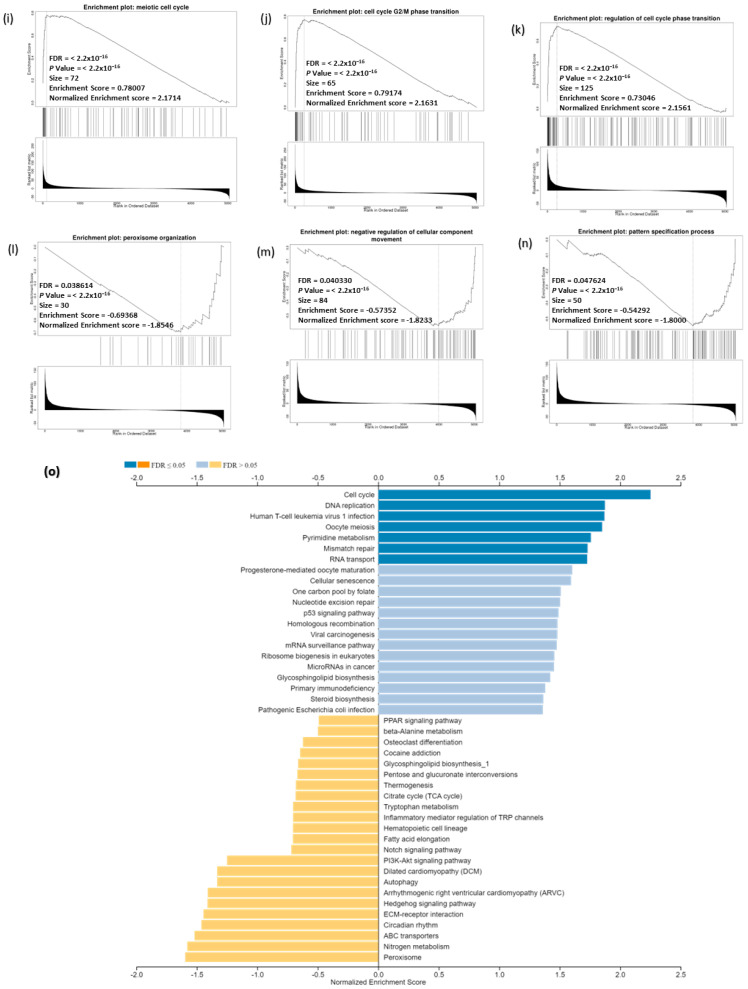

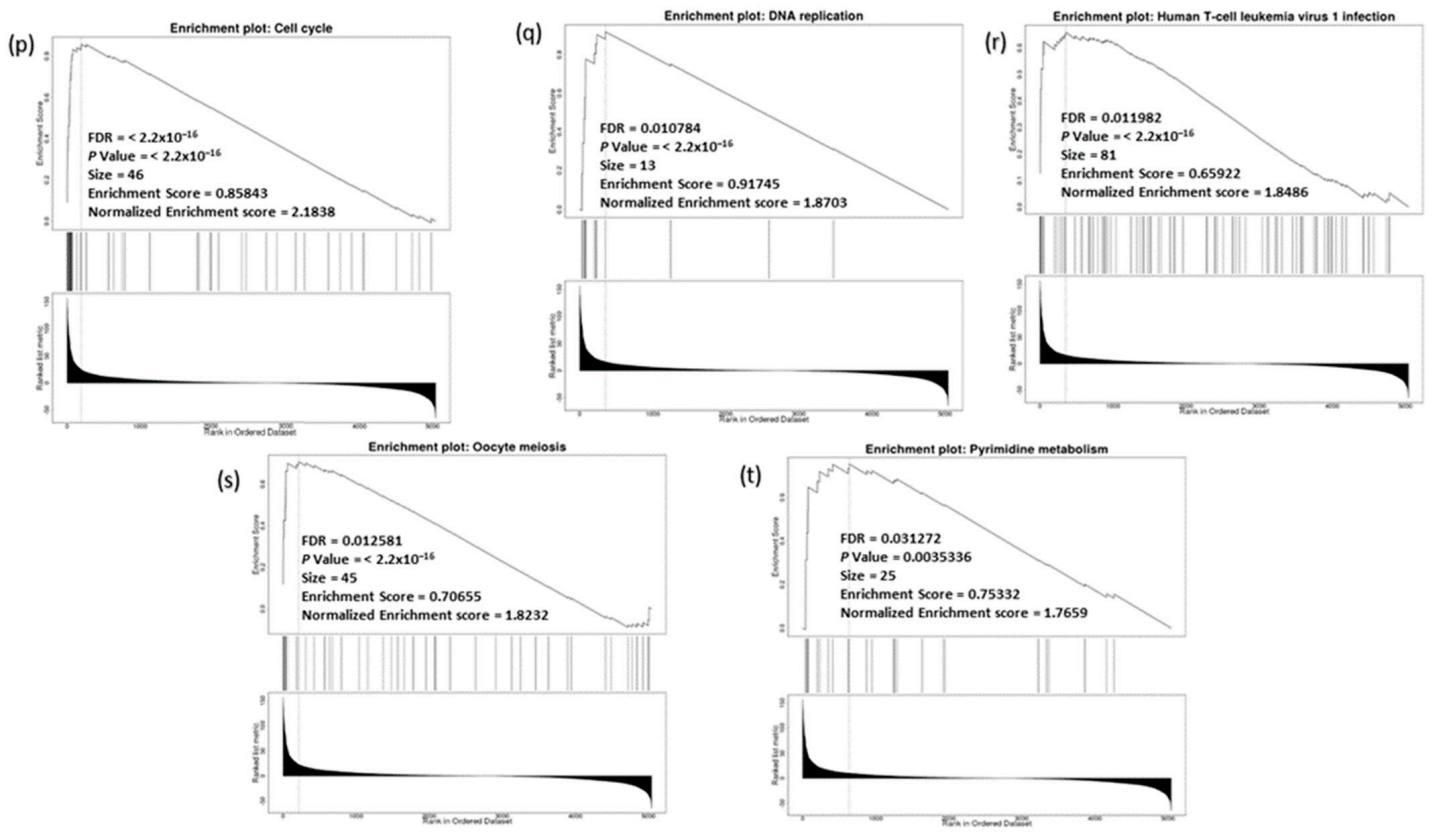
Analysis of GO and KEGG biological pathways of the genes co-expressed with CDC20 in BC. (**a**) Volcano plots for the genes that were positively (red dots) or negatively correlated (green dots) with CDC20 expression. The red and green arrows indicated a positive or negative correlation. (**b**) GO chart bar analysis for the biological process of controlling genes co-expressing with CDC20: positively (blue bars) and negatively (orange boars) correlated gene profiles. (**c**–**n**) The top nine enriched plots of KEGG pathway enriched analysis. (**o**) The enriched biological pathways identification using GSEA. (**p**–**t**) The most significant pathways that were over-represented among negatively correlated genes. FDR and *p* values ˂ 0.05 are statistically significant. GO; Gene Ontology, KEGG; Kyoto Encyclopedia of Genes and Genomes database, GSEA; Gene Set Enrichment Analysis.

To identify genes related to CDC20 expression in BC, we used different modules of LinkedOmics: LinkFinder and Link Interpreter module. The GSEA tool under the Link Interpreter module generated GO and KEGG biological pathways data for CDC20-related genes (Figure 11). The volcano plot of Spearman’s rho statistics (Figure 11a) incorporated the genes associated with CDC20 expression, either positively (red) or negatively (green). The chart demonstrated numerous upregulated genes linked to CDC20 expression, with the BUB1 gene being the most co-expressed among other biological processes-related genes, followed by CDCA3. On the contrary, LOC492311 is the most negatively co-expressed with CDC20. The chart bars for GO (Figure 11b) profiles biological processes related to the genes that co-express with CDC20 positively (blue bars) or negatively (orange bars). The pathways corresponding to genes that CDC20 inversely co-expressed were enriched significantly (*p* ˂ 0.05 and false discovery rate, FDR *p* ˂ 0.05). The GO results indicated that chromosome segregation, organelle fission, and mitotic cell cycle phase transition were the most positively related biological processes to CDC20 overexpression. In contrast, pigmentation, pattern specification process, and negative regulation of cellular component movement were the most repressed processes with their negative correlation to CDC20). Furthermore, the top nine inversely co-expressed genes were presented on enriched plots (Figure 11c–k) as enriched biological pathways as follows: the chromosome segregation (GO: 0007059), mitotic cell cycle phase transition (GO:0044772), organelle fission (GO: 0048285), spindle organization (GO: (GO:0007051), DNA replication (GO:0006260), microtubule cytoskeleton organization involved in mitosis (GO:1902850), meiotic cell cycle (GO:0051321), cell cycle G2/M phase transition (GO:0044839), and regulation of cell cycle phase transition (GO:1901987) pathways, respectively. On the contrary, the three most significant CDC20-positively correlated gene-enriched pathways were pattern specification process (GO:0007389), peroxisome organization (GO:0007031), negative regulation of cellular component movement (GO:0051271), respectively (Figure 11l–n).

The GSEA tool was also used to probe the KEGG database and identify the enriched biological pathways (Figure 11o). The significant pathways enrichment was again found in the cell cycle (hsa04110) and DNA replication (hsa03030), in addition to other related pathways including, human T-cell leukemia virus 1 infection (hsa05166), oocyte meiosis (hsa04114), pyrimidine metabolism (hsa00240) (Figure 11p–t). Interestingly, no significant enriched pathways were recognized for genes positively co-expressed with CDC20.

**Figure 12:**
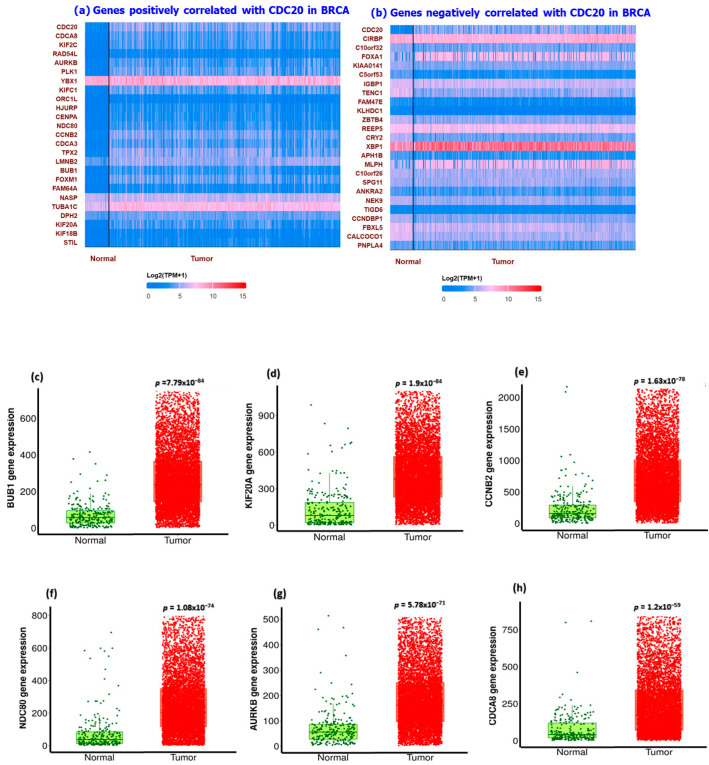

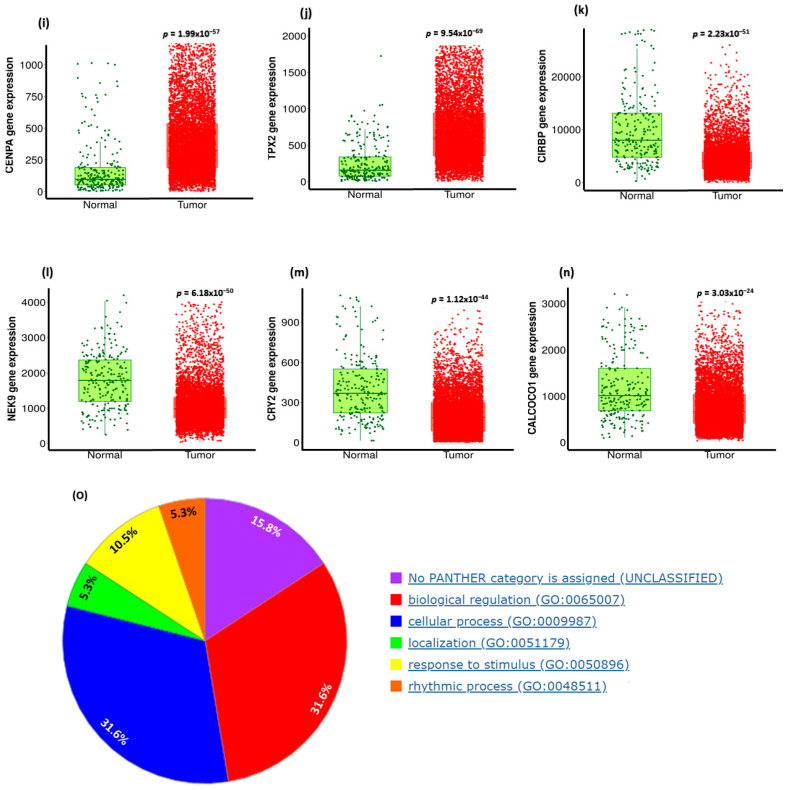
Summary of CDC20-related gene expression and functional classification in BC. Heatmaps of genes with (**a**) positive correlation with CDC20 and (**b**) negative correlation with CDC20. Compared with the control, (**c**–**j**) were the most significantly upregulated genes, and (**k**–**n**) were the most downregulated genes that mediated cancer progression and carcinogenesis. (**o**) A pie chart generated using PANTHER to identify different target gene-involved biological processes highlighted biological regulation and cellular processes as the most biological processes related to CDC20 expression.

We utilized UALCAN and PANTHER databases to explore and functionally classify the genes correlated with CDC20 expression in BC (Figure 12). In relation to CDC20 expression, heatmaps showed the most related genes positive (YBX1 and TUBA1C) or negative (XBP1 and CIRSP) (Figure 12a,b). In comparison with the normal breast tissues, the most upregulated eight genes in BC tissues, with the corresponding fold-change, are shown in Figure 12c–j as follows: UB1 (4.05-folds), KIF20A (4.86-folds), CCNB2 (4.03-folds), NDC80 (5.79-folds), AURKB (2.98-folds), CDCA8 (4.57-folds), CENPA (3.51-folds), and TPX2 (3.94-folds). The top four significantly downregulated genes were CIRBP, NEK9, CRY2, and CALCOCO1 (Figure 12k–n). Another tool, PANTHER, was accessed to identify and classify the different biological processes controlled by these genes. The generated data presented in the pie chart indicated that the biological regulation (31%) and cellular process (31.6%) genes were the most prevalent genes associated with CDC20 expression in BC (Figure 12o).

### 3.10. TNBC IHC Analysis

**Figure 13:**
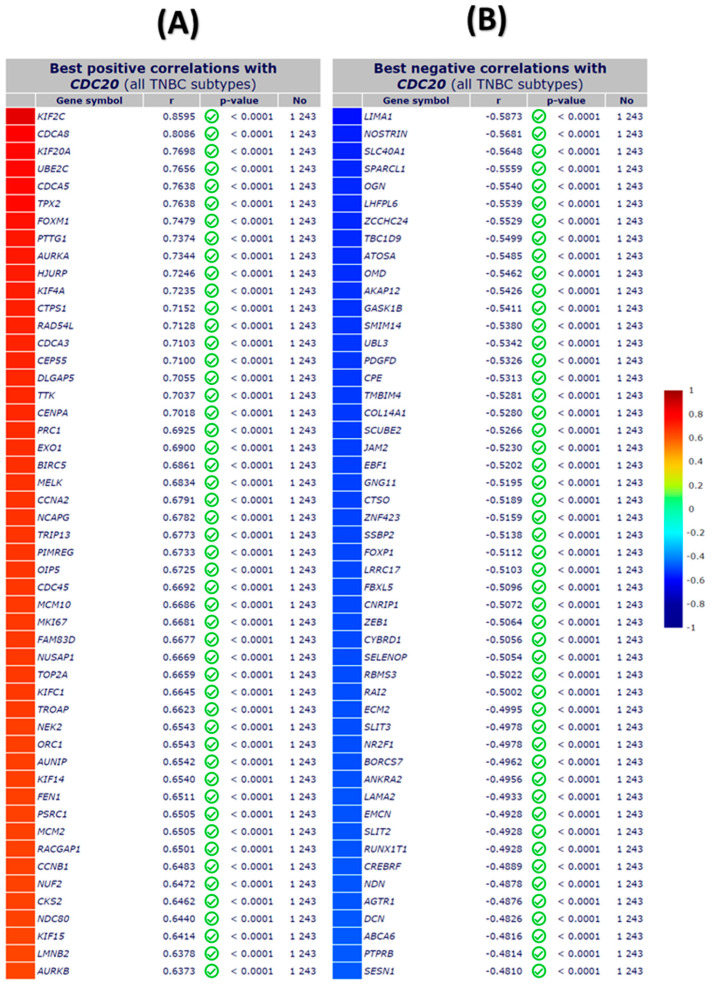
The top fifty genes with positive and negative correlation with CDC20 in TNBC using IHC microarray analysis. (**A**) The top positive correlation with CDC20, and (**B**) the top negative correlation with CDC20. All DNA microarray data for gene expression correlation analyses were generated from the gene correlation exhaustive analysis module bc-GenExMiner using the TIMER database.

In the investigation of TNBC cells, a microarray of genes using immunochemistry (IHC) and bc-GenExMiner module was employed to explore the most positively and negatively correlated gene expression with CDC20 (Figure 13). The gradient color scale on the right demonstrated the score codes for the top fifty genes, with +/− r values of correlation, respectively. Among those positively linked to CDC20, we found KIF2C and CDCA8 the top two genes on the list (r = 0.8595, 0.8086, respectively), whereas LMNB2 and AURKB were the least (r = 0.6378 and 0.6373, respectively) (Figure 13A). On the other hand, the negative correlation with CDC20 presented in Figure 13B denoted LIMA1 (r = −0.5873) and SESN1 (r = −0.481) as the highest and lowest negatively correlated gene, respectively.

### 3.11. CDC20 Is Correlated with Tumor Purity and Immune Infiltration Levels in BC

**Figure 14:**
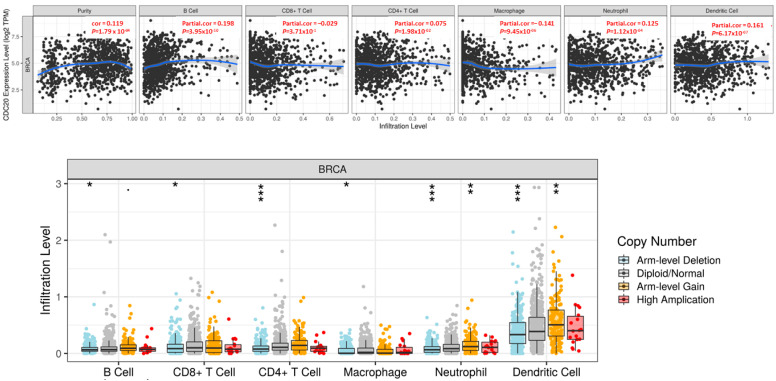
Correlation between CDC20 gene expression and infiltration levels of immune cells in invasive BC. The upper graph: The CDC20 expression correlated positively with tumor purity and the infiltrating levels of B cells, CD4+ T cells, neutrophils, and dendritic cells. A negative association with CDC20 was only found in the infiltration level of macrophage. The lower graph: CNV of CDC20 indicated a significant effect in the infiltration level of all tested immune cells in BC. *p*-value; 0 ≤ *** < 0.001 ≤ ** < 0.01 ≤ * < 0.05 ≤ 0.1. CNV; copy number variation.

We used the TIMER database to investigate whether CDC20 expression correlates with the immune cells’ infiltration level in BC tissues. The generated scatter plots showed the purity-corrected partial Spearman’s rho value and statistical significance, as displayed in Figure 14. The CDC20 expression level in BC exhibited a significant positive link (*p* = 1.79 × 10^−04^) to tumor purity, the ratio of cancer cells in a sample. Also, the correlation between the mRNA expression of CDC20 and infiltrating immune cells in BC showed a significant positive association with a plethora of B cells (cor = 0.198, *p* = 3.95 × 10^−10^), CD4+ T cells (cor = 0.075, *p* = 1.98 × 10^−02^), neutrophils (cor = 0.125, *p* = 1.2 × 10^−04^), and dendritic cells (cor = 0.161, *p* = 6.17 × 10^−07^). There was no significant correlation to CD8+ T cells (cor = −0.029, *p* = 3.71 × 10^−01^). On the other hand, a significant negative correlation was only exhibited between CDC20 expression and the infiltration level of macrophages (cor = −0.141, *p* = 9.45 × 10^−06^). These findings emphasize the regulatory role of CDC20 expression in modulating the infiltration level of immune cells in BC tissues.

Furthermore, Figure 14 shows the association between SCNA of the CDC20 gene with immune cell infiltration in invasive BC. SCNAs, a pervasive trait of human cancer cells, are defined by GISTIC 2.0 and include arm-level deletion, diploid/normal, arm-level gain, and high amplification. The generated SCNA of CDC20 had a significant negative correlation with infiltration levels in all immune cells.

### 3.12. Pan-Cancer Analysis

**Figure 15:**
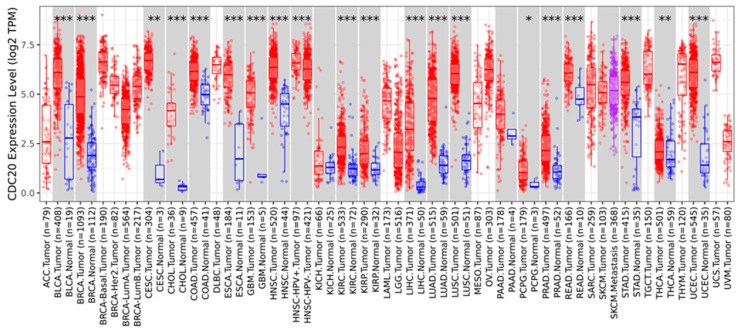
Expression profile of CDC20 in several types of cancer. Distributions of gene expression levels compared to normal tissues and boxplots of up- and downregulated genes for each cancer type in gray columns when normal data were available. Data were generated using the TIMER 2.0 database. BLCA; bladder urothelial carcinomas, BC; breast invasive carcinoma, CHOL; cholangiocarcinoma, COAD; colon adenocarcinoma, ESCA; esophageal carcinomas, GBM; glioblastoma multiforme, HNSC; head–neck squamous cell carcinoma, HNSC-HPV+; head–neck squamous cell carcinoma–human papillomavirus+, HNSC-HPV-; head–neck squamous cell carcinoma–human papillomavirus-, KIRC; kidney renal clear cell carcinomas, KIRP; kidney renal papillary cell carcinomas, LIHC; liver hepatocellular carcinoma, LUAD; lung adenocarcinoma, LUSC; lung squamous cell carcinomas, PRAD; prostate adenocarcinoma, READ; rectum adenocarcinoma, STAD; stomach adenocarcinomas, UCEC; uterine corpus endometrial carcinoma, CESC; cervical squamous cell carcinoma, THCA; thyroid carcinomas, PCPG; pheochromocytoma and paraganglioma, KICH; kidney chromophobe, SKCM; skin cutaneous melanoma cancers. *p*-value; * < 0.05, ** < 0.01, *** < 0.001.

According to the TCGA database, we evaluated CDC20 expression levels in various cancer types. As demonstrated in Figure 15, the expression level of CDC20 was significant (*p* < 0.05, *p <* 0.01, and *p* < 0.001 Figure 15 and Table 2). Compared to the corresponding normal tissue, a highly significant expression (*p* < 0.001) was found in eighteen tumor tissues, including bladder urothelial carcinomas (BLCA), invasive breast carcinoma (BC), cholangiocarcinomas (CHOL), colon adenocarcinoma (COAD), esophageal carcinomas (ESCA), glioblastoma multiforme (GBM), squamous cell carcinoma (SCC) of the head and neck (HNSC), Human Papilloma Virus (HPV)+ SCC of the head and neck (HNSC-HPV+), HPV- SCC of the head and neck (HNSC-HPV-), kidney renal clear cell carcinomas (KIRC), kidney renal papillary cell carcinomas (KIRP), liver hepatocellular carcinoma (LIHC), lung adenocarcinoma (LUAD), lung squamous cell carcinomas (LUSC), prostate adenocarcinoma (PRAD), rectum adenocarcinoma (READ), stomach adenocarcinomas (STAD), and uterine corpus endometrial carcinoma (UCEC). A lower expression (*p* < 0.01) was also found in tumor tissues of cervical squamous cell carcinoma (CESC) and thyroid carcinomas (THCA) as well as pheochromocytoma and paraganglioma (PCPG, *p* < 0.01). Meanwhile, no significant upregulated expression was shown for kidney chromophobe (KICH) and skin cutaneous melanoma (SKCM) cancers.

### 3.13. CDC20 Linked Gene Enrichment Analysis Data

**Figure 16:**
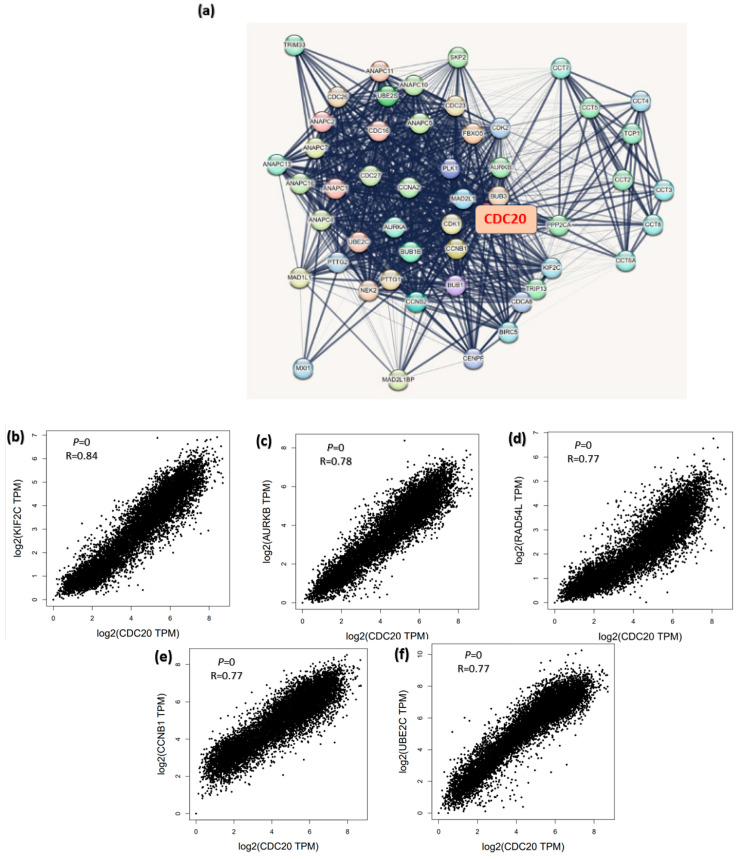

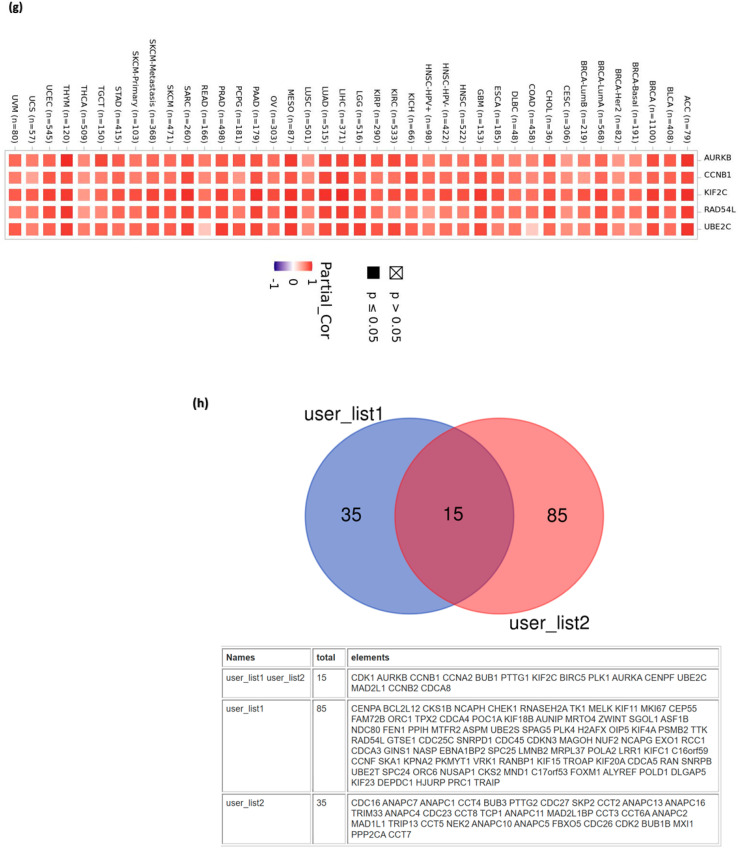
CDC20-related genes enrichment analysis. (**a**) The CDC20-binding proteins were identified using the STRING tool. (**b**–**f**) The correlation between the expression of CDC20 and the top five genes co-expressed with CDC20: KIF2C, AURKB, RAD54L, CCNB1, and UBE2C. (**g**) Heatmap displayed the correlation between the expression of CDC20 and the top five genes’ co-expression with CDC20, KIF2C, AURKB, RAD54L, CCNB1, and UBE2C) in the detailed cancer types. (**h**) Venn diagram illustrating the intersection analysis of the CDC20-binding protein and correlated genes.

To study how CDC20 gene expression is related to tumorigenesis, we screened CDC20-binding proteins and CDC20 expression-related genes. A series of pathway and function enrichment analyses were conducted. The STRING tool was used to screen the top fifty CDC20 binding proteins. Figure 16a shows the interaction network of these fifty proteins. Next, we obtained the top 100 genes related to CDC20 expression using all tumor expression databases of TCGA and the GEPIA2 tool. As shown in Figure 16b–f, the expression level of CDC20 was significantly (*p* = 0.0) and positively correlated with genes encoding Kinesin Family Member 2C (KIF2C; r = 0.84), Aurora Kinase B (AURKB; r = 0.78), RAD54 Like (RAD54L; r = 0.77), Cyclin B1 (CCNB1; r = 0.77), Ubiquitin Conjugating Enzyme E2C (UBE2C; r = 0.77). The corresponding heatmap data (Figure 16g) also showed that the expression level of CDC20 was positively correlated with the above five genes in most tumor types. The top 50 CDC20 binding proteins and the top 100 CDC20 expression-linked-genes were cross-analyzed to generate fifteen common genes, presented in the Venn diagram (Figure 16h).

## 4. Discussion

Innovative methods and early detection have demonstrated a significant impact on lowering death rates across a range of cancer types [29]. Cancer genomics is continuously growing to interrogate cancer’s cellular and molecular details. Various molecules serve as potential biomarkers, offering unique insights into several aspects of biological processes. BC biomarkers are being studied, including hormone receptors (ER and PR) and HER2. These biomarkers are measurable indicators that can be used to detect or evaluate the presence or progression of BC. However, searching for new potential biomarkers is continually being investigated. The gene CDC20 has been implicated in various diseases, including cancer. Using integrated bioinformatics analysis, CDC20 has been previously identified as a potential drug target for cholangiocarcinoma (CCA) and hepatocellular carcinoma (HCC) [60,61].

CDC20 is a well-known regulator of cell cycle progression. This normal mechanism is achieved through variable expression levels and different localizations of CDC20 across various stages of the cell cycle [43]. Abnormal overexpression of CDC20 can lead to mitotic defects, the main cause of cancer development. In our study, integrative bioinformatics analysis has been identified as a potential tool for studying CDC20. Investigating the expression of CDC20 in several clinicopathological features using the TCGA database and UALCAN analysis indicated the upregulated level of CDC20 in BC malignant tumors compared to normal tissues (Figure 2), as validated using IHC images. We suggest that these expressive, qualitative IHC images are consistent with the upregulated transcript expression of CDC20 in BC observed in TCGA database analysis.

High expression of the oncogene CDC20 has been demonstrated in several types of human malignancies [38]. Indeed, overexpressed CDC20 at both the genes and protein levels have been previously reported in many cancers, including BC, pancreatic, prostate, colorectal, bladder, and lung cancer [37,39,40,44]. In BC, the oncogenic roles of CDC20 are strongly associated with poor prognosis [14,43]. Among different subtypes of BC, TNBC showed the highest expression (Figure 2). The subtype luminol has the best prognosis among BC subtypes [14]. This notion is consistent with the low CDC20 expression found in this investigation. The current investigation indicated that CDC20 expression at both gene and protein levels is higher in BC patients compared with normal breast tissue. In contrast, TNBC was the most prevalent among different BC subclasses. In further analysis, we found higher levels of CDC20 in the absence of ER, PR, and HER2 receptors in basal and TNBC subtype patients (Figure 3). These results have validated our hypotheses and interpreted the high expression of CDC20 in TNBC cells, holding these characteristics (Figure 3). Also, the upregulated expression of CDC20 in mutated BC1/2 (Figure 3f) was seen in African American women, characterized by incrementally mutated BC1/2 [62,63,64]. These findings may be one of the many factors that lead to the 42% higher mortality rate in African American BC patients compared to their Caucasian and Asian counterparts [65].

A previous study using the BC gene expression database reported an upregulated level of the CDC20 gene in approximately 15,000 TNBC patients [14]. Another database investigation using more than 2000 TNBC patients reported a significantly overexpressed CDC20 in only TNBC compared with other subtypes [14]. Also, the expression of CDC20 was elevated in 19 of a total of 445 BC patient samples, of which TNBC patients were more frequent [44].

From a survival perspective, TCGA and UALCAN data analysis (Figure 4) revealed that TNBC patients of African American origin showed a high expression of CDC20 at all stages of progression in line with the nodal metastasis. This augmented expression is concurrent with BC progression, a poor prognosis, and resistance to endocrine treatment in BC patients receiving hormone therapy alone [66]. In TNBC human samples, CDC20 upregulation was found to augment tumor cell growth, migration, metastasis, and decreased overall survival [14]. These previous findings suggest CDC20 as an independent prognostic biomarker [40].

When comparing the RFS with CPR for all BC subtypes, the expression of the CDC20 oncogene was inversely correlated with patient survival (Figure 5). There was a remarkable decrease in CDC20 expression and an increase in the RFS when BC patients received any chemotherapy or endo therapy. Indeed, the substantial role of elevated CDC20 mRNA expression in BC progression and poor clinicopathological features was previously cited, as it was considerably more prevalent in patients with large tumor sizes and high-grade primary malignant tumors [66,67]. In parallel, upregulated expression of CDC20 was strongly associated with a lower 5-year recurrence-free survival rate [39], the findings that support our analysis and interpret the insignificant RFS (Figure 6) and CPR (Figure 7), shown by TNBC patients when treated with any chemotherapy.

We further investigated the impact of CDC20 overexpression on important clinical endpoints in oncology: DMFS, OS, and DFS. The KM plots of TNBC and its four subtypes; BLIS, BLIA, MLIA, and LAR; Figure 8, showed no significant difference between low and high CDC20 expression to DMFS, OS, or DFS. However, CDC20 deficiency decreased cell growth and reduced metastasis in four TNBC cell lines [14]. Also, the depletion of CDC20 in pancreatic cancer patients suppressed cell growth, induced G2/M cell cycle arrest, boosted the cytotoxic effect of paclitaxel treatment, and enhanced the effect of gamma-irradiation [68]. In line, the knockdown of CDC20 is a promising approach in treating different cancers, including lung [69], prostate [36], colorectal [70], hepatocellular carcinoma [71], and gastric cancers [72], as well as many other types. Hence, this oncogene is considered a potential therapeutic target for various aggressive cancer types [37], and the development of CDC20 inhibitors could be a novel avenue that proceeds from preclinical to clinical studies to treat tumors [43].

Since TNBC encompasses mutated genes controlling cell proliferation, such as BC1/2 [73], we highlighted the impact of the most frequently altered genes on CDC20 expression (Figure 9 and Table 1). TP53, FAT3, RELN, SPTA1, F5, and DYNC2H1 mutants have shown a significant increase in CDC20 transcript levels, with TP53 mutants being the highest among all. Indeed, previous reports have demonstrated the association of these genes to cell cycle and mitosis.

Under normal cellular conditions, TP53 functions as a tumor suppressor by targeting cells with damaged genomes and maintaining the integrity of the genome [74]. However, mutant TP53 loses these defense mechanisms and starts to trigger transcription of crucial genes primarily involved in cell cycle arrest, apoptosis, and DNA repair, which allow cancer survival [75]. FAT3 and RELN, well-known tumor suppressors and mutated genes, are closely linked to poor prognosis in various cancer types, including TBNC [76,77,78]. DYNC2H1 is generally downregulated in BC, which is the conception that revokes ciliogenesis and leads to the loss of cilia in BC patients [79]. A case–control study highlighted the correlation between genetic variants in the F5 gene and BC susceptibility [80]. Other studies have linked SPTA1 to colorectal [81] and small cell lung [82] cancers. In BC, overexpression of F5 is implicated in the disease’s aggressive nature and decreased overall survival [83,84]. In contrast, mutant SPTA1 is mutually exclusive to the core members of cell cycle pathways and is suggested to be associated with abnormal cell proliferation in glioblastoma development [85]. In contrast, CDC20 expression is reduced in other mutants; CDH1, PIK3CA, MAP3K1, and GATA3. In BC, these mutant genes are associated with tumor grade and IHC intensity. Hence, a positive association was found between the number of mutated genes and the advanced tumor grades [86].

Since CDC20 is a putative transcription factor in different subtypes of BC, potential target genes were identified by searching the GSEA Molecular Signature Database (Figure 11). We investigated the differential expression of the genes targeted by CDC20 and its role as either tumor suppressor or promoter. In this study, eight oncogenes were significantly upregulated (BUB1, KIF20A, CCNB2, NDC80, AURKB, CDCA8, CENPA, and TPX2). On the other side, four tumor suppressor genes (CIRBP, NEK9, CRY2, and CALCOCO1) were demonstrated. All these genes are components of the cell cycle process. For illustration, the mitotic checkpoint serine/threonine kinase BUB1 [87] is a key factor in mitosis [88], spindle assembly checkpoint, chromosome segregation, and DNA damage response [89]. In BC, BUB1 is essential in preserving cancer stem cells [90], and its upregulation could be a promising prognostic biomarker related to poor prognosis in several cancer types, including BC [91]. Also, the downregulated cold-inducible RNA binding protein CIRBP has been previously shown to play a pro-oncogenic role [92]. The RNA-binding proteins, RBPs, orchestrate post-transcriptional regulation of gene expression and have evolved as essential modulators of cancer progression [93,94]. CIRBP is involved in controlling DNA repair and cell proliferation, and it plays a critical role in several human disorders, including cancer and inflammatory diseases. Despite being primarily thought of as an oncogene, CIRBP may potentially play a part in tumor suppression [92].

In our investigation, GSEA demonstrated the GO and KEGG biological pathways for CDC20-linked genes (Figure 11c–k). Indeed, the results denoted that cell-cycle-related pathways were overrepresented inversely, thus suggesting that CDC20 is significantly controlling the cell cycle and apoptosis in BC cells.

As tumor-infiltrating immune cells play a crucial role in cancer incidence, progression, and metastasis [95], we investigated whether CDC20 expression was correlated with immune infiltration levels in BC using the TIMER database (Figure 13). CDC20 mRNA levels correlated with the different immune cell type markers in BC. This study demonstrates a significant positive correlation between CDC20 expression and the infiltration of B cells, dendritic cells, neutrophils, and CD4+ T cells (Figure 14). The finding suggests that CDC20 plays a fundamental role in controlling tumor immunity and consequently influences BC prognosis.

Given that apoptotic proteins and the cell cycle process are frequently synchronized to preserve tissue homeostasis, inhibiting the expression of both anti-apoptotic and cell cycle proteins simultaneously may result in cell cycle arrest and mitigate the growth of breast cancer cells [96]. Our recently published study highlighted the importance of targeting BIRC5 as a promising tool for BC patients [26]. CDC20 and BIRC5 displayed novel therapy efficacy in BC cells [97]. This advanced research and our bioinformatics analysis would add a piece of evidence to the emerging idea that CDC20 might contribute to BC progression and drug resistance.

A pan-cancer analysis of a specific gene is a promising tool for identifying its general phenotypic characteristics and features and understanding its molecular mechanism and association with prospective clinical prognosis [98]. Therefore, a comprehensive analysis of the functions and molecular mechanisms of CDC20 in pan-cancer is essential to gain advanced insights into relevant cancer mechanisms and enlighten strategies for anticancer drug development. Our study found that CDC20 mRNA expression levels were significantly overexpressed in most human cancers (Figure 15 and Figure 16), as documented in the UALCAN, TCGA, and TIMER2. In line with this, CDC20 overexpression was previously found in many human cancers [99].

Cell cycle defects are the lead of carcinogenesis. This process involves several checkpoints in the cell cycle. Carcinogenesis is associated with the G1/S checkpoint, which integrates the regulation of cell proliferation and differentiation, whereas the mitotic spindle checkpoint is associated with the development of chromosomal instability. Our CDC20 related gene enrichment analysis using STRING and GEPIA2 generated fifteen common genes involved in CDC20 expression: AURKB, CDK1, CCNB1, CCNA2, BUB1, PTTG1, BIRC5, PLK1, AURKA, CENPF, UBE2C, MAD2L1, CCNB2, and CDCA8. These genes are involved in the cell cycle network [36,100]. Additional studies will focus on CDC20 inhibition as a potential therapeutic option for BC patients with CDC20 overexpression.

## 5. Conclusions

**Figure 17:**
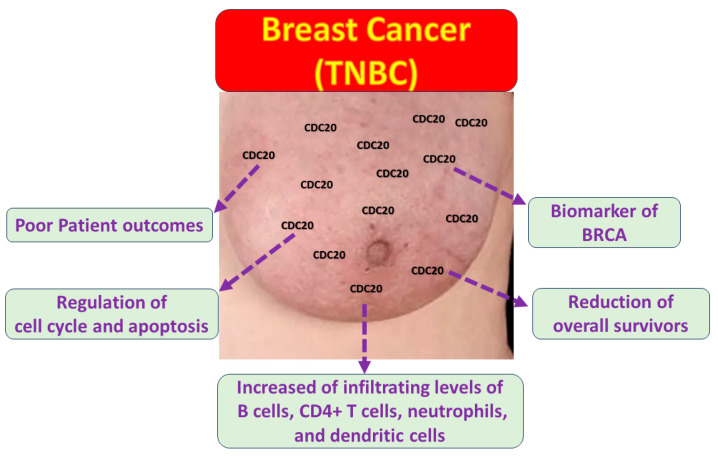
The impact of CDC20 overexpression in TNBC cells.

Breast cancer (BC) is a heterogeneous malignant tumor with several biological subtypes, diverse behaviors, and therapeutic outcomes. The molecular pathogenesis of BC is still poorly definite. Therefore, prospecting the transcriptomic and proteomic expression of the genetic pathways mediating invasion and metastasis is a key factor in enhancing clinical management. The advanced BC database allowed researchers to characterize different parameters such as DNA, gene expression, proteins, and molecular features that may lead to cancer. CDC20 is overexpressed in diverse types of human tumors. The upregulated level of CDC20 is linked to cancer initiation, progression, and metastasis, as well as aggressive behavior and chemotherapy resistance in BC, particularly the TNBC subtype. In the current study, we showed outstanding high expression of CDC20 in tumors compared to normal tissues. This overexpression is linked with clinical stage, metastasis, and decreased overall survival in BC patients, particularly TNBC. Our functional enrichment analysis demonstrated the significant association of CDC20 co-expressed genes with biological regulation and cellular processes. We also found a significant positive correlation between overexpressed CDC20 and tumor purity and many immune cells; the finding suggests that CDC20 plays a fundamental role in controlling tumor immunity and consequently influences BC prognosis (Figure 17). As a potential therapeutic target and a biomarker of many human cancers, developing natural and synthetic inhibitors of this oncogene is a promising approach that could proceed from preclinical to clinical studies. Indeed, CDC20 deficiency led to decreased cell growth and metastasis, G2/M cell cycle arrest, and boosted the cytotoxic effects of paclitaxel treatment, which supported the current analysis. Accordingly, CDC20 could be a legitimate target of medication development for treating BC and other human malignancies.

## Figures and Tables

**Table 1:** Summary of the commonly altered genes that significantly impact the expression of CDC20. The data were used from muTarget analysis tool.

Mutation of	Mean Expression (Mutant)	Mean Expression (Wild)	Mutant Number	Wild Number	FC (Mutant/Wild)	Direction	*p*-Value
TP53	1975.97	693.98	336	643	2.85	up	2.5 × 10^−67^
CDH1	566.91	1227.02	138	841	2.17	down	3.55 × 10^−13^
PIK3CA	774.27	1311.07	323	656	1.69	down	1.02 × 10^−12^
MAP3K1	657.46	1176.37	80	899	1.79	down	1.29 × 10^−5^
FAT3	1827.86	1101.31	44	935	1.66	up	4.06 × 10^−5^
R	1782.88	1112.04	32	947	1.6	up	5 × 10^−5^
SPTA1	1919.39	1088.12	54	925	1.76	up	1.53 × 10^−4^
GATA3	709.19	1198.43	129	850	1.69	down	1.6 × 10^−4^
F5	1708.62	1116.43	29	950	1.53	up	1.82 × 10^−4^
DYNC2H1	1964.19	1105.91	32	947	1.78	up	1.85 × 10^−4^

**Table 2:** Expression profile of CDC20 in different cancer types. Values were obtained from TIMER 2.0, and the threshold *p* ˂ 0.05 was set as a statistically significant value.

Tumor	Normal	Expression	*p*-Value
BLCA. Tumor (*n* = 408)	BLCA. Normal (*n* = 19)	Upregulation	3.5 × 10^−10^
BC. Tumor (*n* = 1093)	BC. Normal (*n* = 112)	Upregulation	1.81 × 10^−58^
CESC. Tumor (*n* = 304)	CESC. Normal (*n* = 3)	Upregulation	2.9 × 10^−3^
CHOL. Tumor (*n* = 36)	CHOL. Normal (*n* = 9)	Upregulation	2.26 × 10^−9^
COAD. Tumor (*n* = 457)	COAD. Normal (*n* = 41)	Upregulation	7.13 × 10^−17^
ESCA. Tumor (*n* = 184)	ESCA. Normal (*n* = 11)	Upregulation	4.11 × 10^−8^
GBM. Tumor (*n* = 153)	GBM. Normal (*n* = 5)	Upregulation	4.3 × 10^−4^
HNSC-HPV+. Tumor (*n* = 97)	HNSC-HPV-. Tumor (*n* = 421)	Upregulation	1.4 × 10^−5^
HNSC. Tumor (*n* = 520)	HNSC. Normal (*n* = 44)	Upregulation	4.34 × 10^−24^
KICH. Tumor (*n* = 66)	KICH. Normal (*n* = 25)	Upregulation	0.343689
KIRC. Tumor (*n* = 533)	KIRC. Normal (*n* = 72)	Upregulation	3.24 × 10^−26^
KIRP. Tumor (*n* = 290)	KIRP. Normal (*n* = 32)	Upregulation	1.43 × 10^−8^
LIHC. Tumor (*n* = 371)	LIHC. Normal (*n* = 50)	Upregulation	5.68 × 10^−28^
LUAD. Tumor (*n* = 515)	LUAD. Normal (*n* = 59)	Upregulation	1.96 × 10^−34^
LUSC. Tumor (*n* = 501)	LUSC. Normal (*n* = 51)	Upregulation	1.18 × 10^−31^
PAAD. Tumor (*n* = 178)	PAAD. Normal (*n* = 4)	Upregulation	6.897 × 10^−2^
PCPG. Tumor (*n* = 179)	PCPG. Normal (*n* = 3)	Upregulation	2.787 × 10^−2^
PRAD. Tumor (*n* = 497)	PRAD. Normal (*n* = 52)	Upregulation	1.21 × 10^−17^
READ. Tumor (*n* = 166)	READ. Normal (*n* = 10)	Upregulation	9.07 × 10^−5^
SKCM. Tumor (*n* = 103)	SKCM. Metastasis (*n* = 368)	Upregulation	0.313568
STAD. Tumor (*n* = 415)	STAD. Normal (*n* = 35)	Upregulation	2.37 × 10^−15^
THCA. Tumor (*n* = 501)	THCA. Normal (*n* = 59)	Upregulation	3.99 × 10^−3^
UCEC. Tumor (*n* = 545)	UCEC. Normal (*n* = 35)	Upregulation	7.21 × 10^−22^

## Data Availability

All data generated or analyzed during this study are included in this published article.
